# Prediction and phenotyping of long COVID in kidney transplant recipients: A cross-sectional study 

**DOI:** 10.5414/CN111584

**Published:** 2025-03-25

**Authors:** Ivan Zahradka, Vojtech Petr, Istvan Modos, Katarina Jakubov, Lukas Dolezal, Szabolcs Kalina, Ondrej Viklicky

**Affiliations:** 1 Department of Nephrology,; 2 Department of Data Science,; 3 Transplant Laboratory, Institute for Clinical and Experimental Medicine, Prague, Czech Republic; *These authors have contributed equally to this work and share the first authorship.

**Keywords:** COVID-19, long COVID, kidney transplantation, phenotypes, symptoms

## Abstract

Aims: The aim was to describe the epidemiology, phenotyping, and risk factors of long COVID (LC) in a well-defined cohort of kidney transplant recipients (KTRs) using a novel LC diagnostic method based on self-reported symptoms. Materials and methods: We conducted a cross-sectional study using an electronic survey to inquire about persisting symptoms following COVID-19. KTRs who survived COVID-19 up to February 8, 2023, were considered for inclusion, and 596 KTRs were enrolled. A brief 35-question screening questionnaire was used. A novel statistical approach based on the factor analysis method was used to make LC diagnosis and identify its clinical phenotypes. Results: LC was identified in 33.7% of KTRs who responded to the survey. Male sex (OR 0.69, 95% CI 0.48 – 1.0, p = 0.047), more severe COVID-19 (OR 2.48, 95% CI 1.58 – 3.92, p < 0.001), higher body mass index (OR 1.04, 95% CI 1.0 – 1.08, p = 0.031), and corticosteroids (OR 2.8, 95% CI 1.23 – 7.09, p = 0.02) were independently associated with LC development. Eight LC phenotypes were identified based on symptom clustering: fatigue (32.4% of all KTRs), psychiatric (15.9%), cardiovascular (6%), ophthalmic (13.8%), cognitive (17.8%), fibromyalgia-like (11.1%), integumental (10.6%), and malnutritional (6%). The rate of LC was similar in those who had COVID-19 less/more than a year since responding to the survey. Conclusion: A novel method for determining LC diagnosis and its phenotyping was used in a large cohort of KTRs, which showed that a third of KTRs who responded to the survey developed LC after COVID-19. This method may improve diagnosis and future research of LC.

## Introduction 

The long-lasting complications of COVID-19 have recently been gaining increasing attention. The various clinical symptoms that affect many COVID-19 survivors are often collectively called long COVID. 

Long COVID is still poorly defined owing to its variable, multisystemic manifestation, and broad and rather vague definition [1]. There is currently no gold standard test for long COVID, therefore the diagnosis is based mainly on self-reporting of subjective symptoms [2]. This leads to significant diagnostic uncertainty and risk of misdiagnosis, which complicates both patient management and enrollment of homogenous patient cohorts to clinical studies. Clearly, better and more objective methods of diagnosing long COVID are one of the currently unmet needs. 

Furthermore, the pathogenesis and incidence of long COVID remain equally poorly understood. Multiple hypotheses such as persistent organ damage, post-intensive care syndrome, persistent tissue viral replication, immune dysregulation, reactivation of latent pathogens, autoimmunity or the induction of myalgic encephalomyelitis/chronic fatigue syndrome (ME/CFS) were suggested [3]. Concurrently, more than 200 different long COVID symptoms have been described, affecting most organs in the body [2, 4, 5]. Therefore, long COVID likely encompasses several different phenotypes or diseases, rather than being a single entity. 

Kidney transplant recipients (KTRs) represent a well-suited cohort for long COVID research. KTRs are among the most vulnerable populations that share many reported risk factors of long COVID development such as frequent underlying comorbidities and impaired immune response [3]. It has already been suggested that the occurrence of long COVID might be higher in KTRs [6, 7] than in the general population [2, 3, 8]. This makes studying long COVID in KTRs both mechanistically and clinically important. 

In this single-center cross-sectional study, we evaluate long COVID symptoms in a large cohort of KTRs with the aim to describe the frequency of persistent symptoms after COVID-19 infection, risk factors of their development, and evaluate whether distinct disease phenotypes can be identified. 

## Materials and methods 

This is a cross-sectional study that aimed to explore the extent, severity, and phenotypes of long COVID among a large cohort of KTRs. The presence of long COVID was evaluated using a statistical method based on the unsupervised factor analysis performed on patient responses to the survey (see Statistics section). The primary aim was to describe the spectrum of long COVID symptoms and their prevalence in KTRs. Among the secondary aims was to identify the risk factors of long COVID development and explore and define long COVID phenotypes. This study was approved by the institutional review board under the reference number A-23-16. 

### Patients 

All KTRs followed at our center who previously survived COVID-19 were considered for inclusion. Patients’ enrollment was based on our prospectively run COVID-19 clinical database where records of all COVID-19 cases in KTRs followed at our center were gathered [9]. Furthermore, to ensure data completeness, the National Registry for Infectious Diseases, where all SARS-CoV-2 tests were mandatorily reported, was also searched [10]. 

All previously SARS-CoV-2-infected KTRs who consented to electronic communication with the transplant center were asked to fill in an online questionnaire with a unique identifier which allowed us to directly connect the answers with our medical records. The questionnaires were collected between December 12, 2022, and February 8, 2023. The patients could answer the questionnaire through their computer or a mobile device (Supplemental Figure 1). 

### Presumed virus variant 

The presumed virus strain causing the COVID-19 infection was assigned to each patient. As viral RNA sequencing was performed only in a minority of cases, the assignment was based on the dominating (expected) virus variant at the time of infection. The wild-type period ranged from March 1, 2020, to January 31, 2021; the alpha period ranged from February 1, 2021, to September 30, 2021; the delta period ranged from October 1, 2021, to December 31, 2021; and the omicron variant was dominant from January 1, 2022, onwards. In the case of reinfections (83 KTRs, 13.9%), only the last infection was considered. 

### Statistics 

The statistical analysis was done in R language version 4.2.2. For factor analysis, we used packages *parameters* [11] and *psych* [12]. For descriptive statistics, we used either Wilcoxon test for ordinal/continuous variables or Fisher’s exact test for categorical variables. 

### Factor analysis 

Factor analysis reveals latent variables (called factors) in the data. By analyzing the coefficients of the question responses (called factor loadings), we can understand the relation between factors and question responses; larger factor loadings represent the higher strength of the relationship. When a set of question responses has high factor loadings with a specific factor, then this factor represents a category (or phenotype) of long COVID. 

Factor analysis suitability was assessed using Kaiser, Meyer, Olkin measure (0.91) and Bartlett’s test (p ≤ 0.001) with Spearman correlation; both suggesting that factor analysis can be applied. Next, to determine the number of factors we used function n_factors from *parameters* package [11] to give us the median number of factors from voting among various methods. To ensure the robustness of the selected number of factors to randomness, the optimal number of selected factors was determined by bootstrapping (50 iterations); the number of factors that occurred most frequently among bootstrapped datasets (8 factors) was chosen as the optimal number (Supplemental Figure 2). Eight factors were then extracted from the whole cohort of 596 patients. The total cumulative explained variance of the selected factors is 43%. The factor analysis itself was done using *psych* package (principal axes algorithm, VARIMAX rotation). We then looked at the factor loadings of responses to each question over all factors and assigned that question responses to a factor with the largest loading; question responses with absolute loading values below 0.3 were ignored. The threshold was set such that the selected questions represent a clinically interpretable factor (for a heatmap of loadings see Supplemental Figure 3). This way, we categorized the questions into different groups, and by analyzing what these questions have in common, we assigned the groups a specific name corresponding to a long COVID phenotype (for instance, fatigue phenotype, cardiovascular phenotype, etc.). In short, all long COVID phenotypes were identified solely on the basis of factor analysis, there was no previous clinical definition. Individual phenotypes were then named according to the predominant symptoms for that phenotype. 

### Long COVID prediction 

First, we computed a factor score for each patient and factor (long COVID type, i.e., fatigue, cardiovascular, etc.). Factor score is obtained by summing the answers to the selected questions within each factor. Next, we trained a multivariable logistic regression model, where the independent variables are the factor scores, and the outcome is the binary answer to the question of significant health deterioration (Question 34; Supplemental Material 1). The whole cohort of 596 patients was used for model training. The area under the curve of the model is 91.95% (Supplemental Figure 4). The threshold for long COVID prediction was found by maximizing the Youden’s Index along the ROC of the model (training accuracy 83.72%, sensitivity 87.86%, specificity 82.46%). Finally, the model was validated on a validation cohort with accuracy 85.56%, sensitivity 73.68%, and specificity 88.73%. The validation cohort consisted of 90 KTRs who filled in the same survey but were part of a different long COVID study, the demographics of this group are shown in Supplemental Table 1. 

### Long COVID phenotype assignment 

To determine whether a patient has a specific long COVID phenotype, we first applied the long COVID prediction model to all 596 patients to determine which patients have long COVID. For the patients with predicted long COVID, we assigned a patient to a specific long COVID phenotype if he/she responded positively to the majority of questions (> 50%) representing that phenotype (1 patient can be assigned to multiple phenotypes). 

### Questionnaire 

As no validated screening tool was available at the time, we developed a short questionnaire consisting of 35 questions (the full questionnaire is available in the Supplemental Material in original with an English translation). The questions were aimed to screen the most common symptoms of long COVID, identified by a previous literature search. Patients were asked to identify any newly emerged or aggravated symptoms that occurred following their recovery from COVID-19. The questionnaire could not be submitted with any questions left unanswered. 

## Results 

### Questionnaire return rate and sample representativeness 

966 COVID-19 survivors from the start of the pandemic to the start of the survey were identified in total. From these, 88 KTRs did not consent to electronic communication. Out of 878 consenting KTRs, 630 KTRs returned the questionnaire (71.8% return rate), while 248 did not (189 did not return the questionnaire at all, while 59 had returned it late after enrollment finished and data analysis started). 34 KTRs were excluded as they had COVID-19 less than 3 months prior to the survey. Therefore, the final analysis included 596 KTRs (study flow chart is shown in Supplemental Figure 5). Those who did not participate in the survey (for any reason) were older (median 61 vs. 56 years, p < 0.001), but overall no other differences in demographics were found. Furthermore, a subgroup of those who did not participate in the survey due to previously not having signed informed consent with electronic communication were more likely to be on cyclosporin therapy compared to participants (19 vs. 7%, p < 0.001). The demographics of those who did not fill out the questionnaire or did not consent to electronic communication are given in Supplemental Table 2. 

### Patient demographics

In total, 596 patients were included in the final analysis; the demographics are given in Table 1. 61% of analyzed patients were male, the median age was 56 years, and 20.5% experienced moderate or worse course of COVID-19, however, only 1% experienced critical illness as defined by the NIH [13]. The viral variant causing the initial COVID-19 infection was wild-type in 21%, alpha in 15%, delta in 13%, and omicron in 52% of cases. KTRs were vaccinated with 2 doses of vaccination prior to COVID-19 in 65% of cases, while 51% were vaccinated with three doses. Reinfections occurred in 13.9% of cases. 

### Survey results 

**Figure 1.**
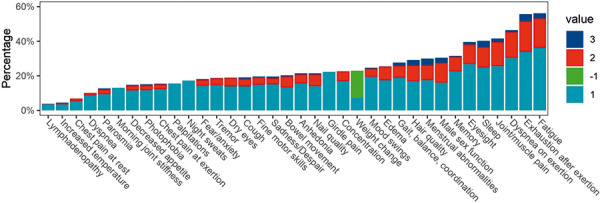
Rates of self-reported long COVID symptoms in the study cohort. Most responses were on a scale: 0 = no symptoms, 1 = mild symptoms, 2 = moderate symptoms, and 3 = severe symptoms. Several yes/no questions: 0 = no, 1 = yes. Unintentional change of body weight question: –1 = decrease, +1 = increase, 0 = no change.

The survey results are summarized in Figure 1. The 10 most commonly reported symptoms were fatigue, exhaustion upon exertion, dyspnea on exertion, joint/muscle pain, disordered sleep, vision impairment, memory impairment/brain fog, decreased hair quality, sexual dysfunction in males or menstrual abnormalities in females of reproductive age. Furthermore, 23.5% of KTRs reported a significant worsening of health and 22% reported a significantly decreased quality of life more than 3 months after COVID-19. 

### Long COVID and its phenotyping 

**Figure 2.**
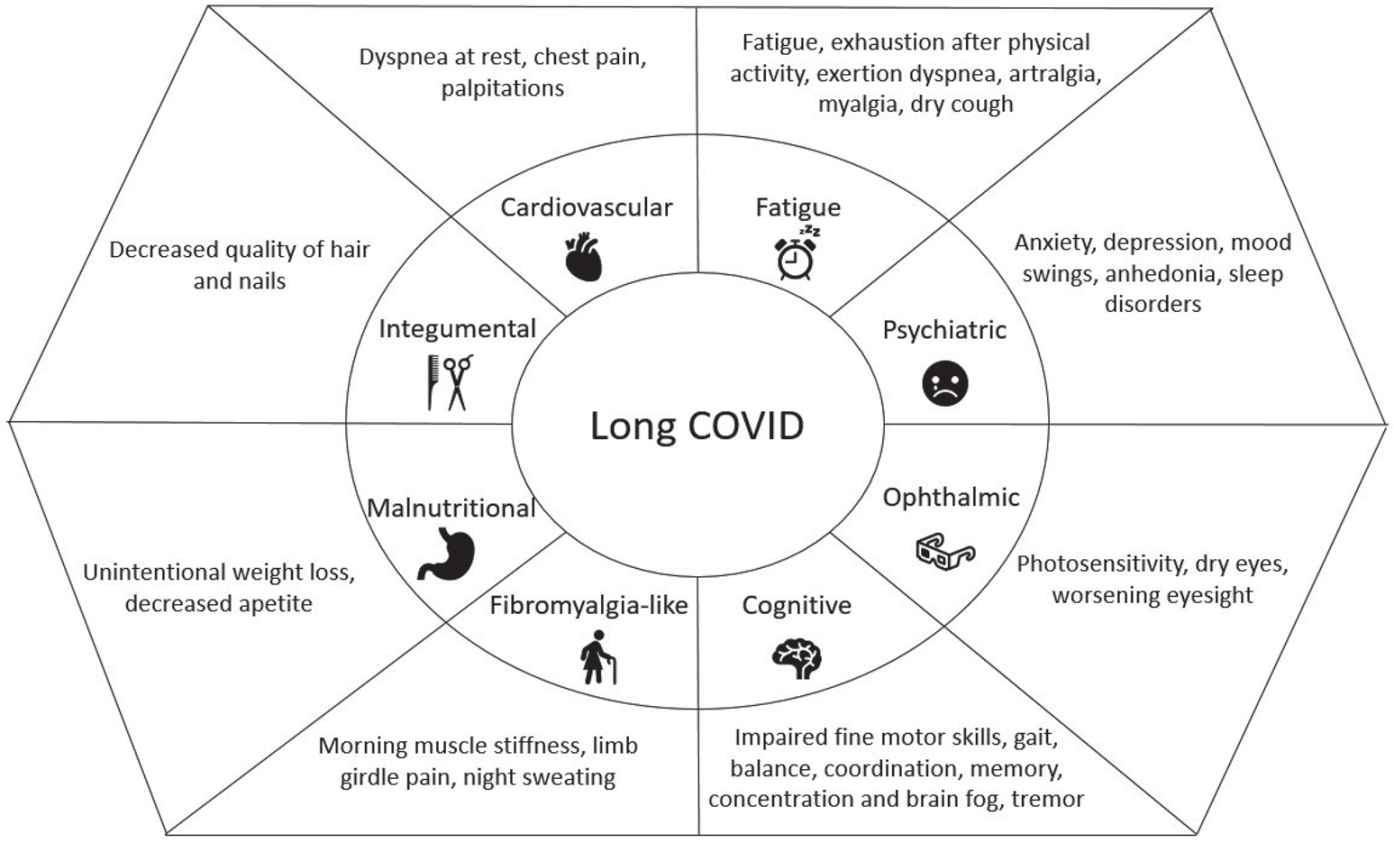
Schematic illustration of long COVID phenotypes. Eight long COVID phenotypes were identified by factor analysis based on clustering of patient self-reported symptoms, defining symptoms for each phenotype are shown.

Next, we performed an unsupervised analysis to categorize patients into long COVID phenotypes based on symptom clustering. Clustering of symptoms that were often reported together, yielded 8 long COVID phenotypes (Supplemental Figure 3). An overview of the individual phenotypes and the defining symptoms are displayed in Figure 2. 

In total, 201 KTRs (33.7%) were designated to have at least 1 long COVID phenotype. The frequencies of individual phenotypes in the cohort were as follows: (1) fatigue (n = 193, 32.4%), (2) psychiatric (n = 95, 15.9%), (3) cardiovascular (n = 36, 6%), (4) ophthalmic (n = 82, 13.8%), (5) cognitive (n = 106, 17.8%), (6) fibromyalgia-like (n = 66, 11.1%), (7) integumental (n = 63, 10.6%), and (8) malnutritional (n = 37, 6%). One patient could have been classified as having more than 1 long COVID phenotype, and indeed many patients developed several long COVID phenotypes, the overlap is depicted in Supplemental Figure 6. Finally, we found no differences in phenotype proportions grouped by COVID-19 periods (Supplemental Table 3), reinfection status (Supplemental Table 4), and vaccination status (Supplemental Table 5). 

### Risk factors of long COVID development 

Next, we calculated a multivariable binary logistic regression model where the dependent variable was the presence of long COVID (i.e., having 1 or more long COVID phenotypes). The results of univariable associations are given in Supplemental Table 6, the multivariable model is shown in Table 2. We found that more severe course of COVID-19 (OR 2.48, 95% CI 1.58 – 3.92, p < 0.001), male sex (OR 0.69, 95% CI 0.48 – 1.0, p = 0.047), higher body mass index (BMI) (OR 1.04, 95% CI 1.0 – 1.08, p = 0.031), and corticosteroid use (OR 2.8, 95% CI 1.23 – 7.09, p = 0.02) were independently associated with higher risk of long COVID development. Hospitalization was not included in multivariable analysis as it was highly colinear with symptom severity, which provides better information as hospitalizations were not always reflective of the patient’s clinical status. Furthermore, patients during the pandemic could have received specific antiviral treatment, namely monoclonal antibodies, molnupiravir, and remdesivir. Paxlovid was not used due to its interaction with tacrolimus. In our analysis, specific antiviral treatment was not associated with lower odds of developing long COVID. 

### Long COVID phenotypes demographics 

When patients with and without long COVID were compared, we found that the long COVID group was older (59 vs. 55 years, p = 0.003), had more frequently moderate or worse COVID-19 (30 vs. 15%, p < 0.001), lower estimated glomerular filtration rate (eGFR) before COVID-19 (46 vs. 51 mL/min/1.73m^2^), higher BMI (28.4 vs. 27.6 kg/m^2^, p = 0.016), and had higher Charlson Comorbidity Index (CCI) (4 vs. 3, p = 0.018) (Supplemental Table 7). 

We then compared demographics of individual long COVID phenotypes to patients without long COVID. Patients with most long COVID phenotypes were usually older and had survived a more serious COVID-19 infection. The integumental and psychiatric phenotypes were more common in females (59 vs. 41%, p < 0.001, and 56 vs. 44%, p = 0.002, respectively). Patients who developed the cognitive, fatigue, fibromyalgia-like, and psychiatric phenotypes had higher BMI (29.5 vs. 27.6 kg/m^2^, p = 0.004, 28.4 vs. 27.6 kg/m^2^, p = 0.009, 29.6 vs. 27.6 kg/m^2^, p < 0.001, 28.6 vs. 27.6, p = 0.035, respectively). Worse allograft function before infection (46.2 vs. 51 mL/min/1.73m^2^, p < 0.001) was found in those who developed the fatigue phenotype. Lastly, a longer time from transplantation was found in patients with the cognitive phenotype (71 vs. 56 months, p = 0.034). For a detailed comparison of characteristics see Supplemental Tables 8 – 15, univariable associations with a specific phenotype development are shown in Supplemental Figures 7 – 14. Furthermore, Spearman correlations between factor scores in each phenotype and clinical factors were calculated and can be seen in Supplemental Figure 15. 

### Changes in frequency of long COVID rate and individual responses with time from symptom onset to survey response 

The change in the pattern of survey responses with time from symptom onset was analyzed. The time from the symptom onset to survey response was split into 6-month periods, a corresponding period was assigned to each KTR, and the frequency of positive answers to each question from the survey was evaluated. Overall, the frequency of positive responses did not change in time except for joint/muscle pain (p = 0.004) and decrease in appetite (p = 0.011) (Supplemental Figure 16, Supplemental Table 16). Finally, there were no significant differences in the overall rate of long COVID nor the individual phenotypes between KTRs who experienced COVID-19 more and less than 1 year prior to taking part in the survey (Supplemental Figure 17). 

## Discussion 

In this study, we propose a new prediction model that could be used to delineate a threshold for the presence of long COVID as well as long COVID phenotyping based on symptom self-reporting. Using this model, we show that 33.7% of KTRs who survived COVID-19 and responded to the survey report persistent symptoms that can be classified into eight phenotypes using unsupervised statistical analysis. We also describe risk factors of long COVID development in KTRs among which is corticosteroid use in maintenance immunosuppression. 

One of the most problematic aspects of long COVID is its definition. The WHO (World Health Organization) defines long COVID as a continuation of symptoms or a development of new ones within 3 months after the initial SARS-CoV-2 infection; the symptoms must last for at least 2 months and must have no other explanation or cause [2]. Therefore, the diagnosis of long COVID is based on self-reported symptoms with reasonable exclusion of other causes. No objective diagnostic criteria, specific biomarkers, or imaging methods are, however, currently available. Thus, as any patient with any symptom following COVID-19 that cannot be explained otherwise should be considered as having long COVID, such a definition of long COVID leads to misdiagnosing and overestimation of prevalence. In fact, in one study, almost 10% of COVID-19 negative controls referred at least one of the core long COVID symptoms [8]. This issue could be at least partly resolved by setting a symptom threshold for long COVID diagnosis. 

Our study sets this threshold using criteria based on two assumptions. First, for a symptom to be considered relevant (as opposed to being just a random reply or a symptom usually not associated with long COVID), it should systematically cluster with other symptoms around an identified cluster/phenotype (for instance cardiovascular or cognitive phenotype identified in this study). Second, the symptoms in question should negatively impact the perception of one’s health. Applying these principles, our statistical model yielded a conservative estimate of long COVID prevalence in KTRs. 

Long COVID is an extremely heterogeneous condition [14] with more than 200 possible symptoms [2, 4, 5] and variable causes [3]. Considering the wide spectrum of possible symptoms, the symptomatologic approach could be a viable option to better define patient subgroups for more focused research and treatment. Several studies have attempted to differentiate long COVID phenotypes, some by utilizing unsupervised methods such as cluster analysis [15, 16, 17, 18, 19, 20, 21, 22, 23], with varying results. Canas et al. [21] employed an unsupervised clustering using k-means to identify symptom profiles for vaccinated and unvaccinated individuals from the general population during different COVID-19 periods. In their study, long COVID was defined as symptoms that persisted 84 days after the initial positive test. The authors found that the optimal number of clusters changed with the period, although the cardiorespiratory, central neurological, and multi-organ systemic inflammatory clusters were present across all variants. Furthermore, no link between vaccination and lower prevalence of long COVID was found. Lastly, there was no control group in this study. In contrast, Bowyer et al. [23] showed only two symptom clusters. One cluster consisted of patients who had common symptoms such as headache or runny nose but in general were feeling well, while the other cluster reflected a high symptom burden. Even though their study had a negative control group, individuals in the negative control group often referred mild to moderate symptoms, which further underlines the problem of false positive cases when self-reporting long COVID symptoms. Taken together, a comparison of our and other studies shows that different statistical methods, type of data collected, and the definition of long COVID itself yield different results. This points to a need to adapt a novel unified approach to self-reporting diagnosis. 

Another question is whether clinically identified phenotypes have any mechanistic or biological foundation. Liew et al. [17] performed blood proteomics in a large long COVID cohort and described distinct proteomic patterns in clinical long COVID phenotypes similar to those described in this study. Liew et al. [17] were therefore one of the first to corroborate the idea that symptom-based phenotyping has also biological or mechanistic foundations. Furthermore, another study identified C2 complement component to be associated with fatigue symptom of long COVID [24]. We believe that these and other studies show that this approach of symptom-based phenotyping is important to navigate future research and therapies of long COVID. 

In our study, we also identified several factors associated with the development of long COVID in KTRs. Most notably, long-term corticosteroid use was associated with an increased risk of developing long COVID in KTRs. Since the majority of KTRs use corticosteroids as part of the maintenance immunosuppression therapy, this could be one of the reasons for the high rate of long COVID in this population. Additionally, corticosteroids are known to prolong viral clearance in influenza and SARS-CoV-2 [25]. Virus persistence was suggested as causal in long COVID development [26, 27]. We note that while the effect of corticosteroids in the statistical modelling is strong, it needs to be considered with caution as 93% of all subjects of the study had corticosteroids in maintenance immunosuppression. 

Some have suggested that long COVID shares similarities with ME/CFS, as around half of the patients with long COVID also meet the criteria for ME/CFS in the general population [3]. In our study, most KTRs who were identified as having long COVID exhibited systematic symptoms consistent with the fatigue phenotype. Surprisingly, some respiratory symptoms including dry cough and dyspnea at exertion were also clustered in this phenotype. 

Importantly, we found no association between SARS-CoV-2 vaccination and risk of long COVID development or a change of its pattern. This is in line with other observations such as the study by Amorim et al. [28], who also found no link between vaccination and lower long COVID rates. However, it is difficult to discern the protective effects of vaccination in the population of KTRs due to many confounding factors. Furthermore, since KTRs exhibit an impaired immune response to vaccination [29, 30], the protective effect is expected to be modest as even the data from the general population are inconclusive [21, 31, 32]. 

The cross-sectional design and use of an unvalidated screening questionnaire are among the limitations. Although validated questionnaires exist, these are more suitable for longitudinal monitoring rather than cross-sectional screening [33]. The questionnaire we used is rather short by design, which is known to promote higher return rates [34, 35, 36]. Another limitation is that our approach might lead to underestimation, as some of the cases with borderline symptomatology might have been labeled by the algorithm as long COVID free. Furthermore, there were 83 patients after COVID-19 reinfection in our cohort. Even though every patient was asked to report symptoms after the last infection, reinfected patients might have not been able to decide when their symptoms began in relation to multiple COVID-19 episodes. However, the rates of long COVID were similar in those infected multiple times when compared to those who filled out the survey after being infected only once. Additionally, we do not have a cohort of KTRs who filled in the questionnaire and were not previously infected by SARS-CoV-2 nor a control group from the general population, and any study using online questionnaires can be affected by reporting bias. Lastly, KTRs can have a multitude of other symptoms and conditions that can be confounded with long COVID and even though we have attempted to take all confounders into account, some residual confounding may remain. 

The strengths are that this was a large single-center cohort with the availability of granular patient data and a high survey return rate. Furthermore, SARS-CoV-2 infections were verified using a government-mandated registry of all COVID-19 cases. 

In conclusion, long COVID and its organ-specific symptom-based phenotypes can be differentiated based on methods using symptom self-reporting. KTRs appear to be at an especially high risk of long COVID, and corticosteroid therapy is associated with its development. Furthermore, we propose a novel statistical framework to predict long COVID. This or a similar approach can be used to guide future research as well as to tailor possible therapeutic interventions. As transplant recipients appear especially vulnerable, the study of long COVID is important to the transplant community. 

## Data availability statement 

Data available on reasonable request from the authors. 

## Authors’ contributions 

All authors were responsible for the conceptualization of the study, participated in the acquisition of resources, and contributed to the review and editing of the final article. IZ, VP, KJ, IM, LD, and SK took part in data curation. IM, IZ, and VP were responsible for formal analysis and validated the results. OV was responsible for funding acquisition. IZ, VP, and KJ conducted the investigation. IZ, VP, IM, and OV were responsible for the methodology. OV was responsible for project administration and supervision. IM, LD, and SK were responsible for the software support. IM took part in the visualization. IZ, VP, and OV wrote the original draft. IZ and VP contributed equally to this work. 

## Funding 

Supported by the Ministry of Health of the Czech Republic grant no. NU22-C-126, its conceptual development of research organizations (Institute for Clinical and Experimental Medicine-IKEM, IN 00023001), and by the project National Institute for Research of Metabolic and Cardiovascular Diseases (Program EXCELES, Project No. LX22NPO5104) – Funded by the European Union – Next Generation EU. 

## Conflict of interest 

The authors declare that they have no known competing financial interests or personal relationships that could have appeared to influence the work reported in this paper. 

**Table 1.** Patient demographics.

Characteristic	Overall (n = 596)
Time between COVID-19 and response, median months (IQR)	11 (9, 22)
Male sex, n (%)	364 (61%)
Age, median (IQR)	56 (47, 67)
COVID-19 severity based on the NIH Clinical Spectrum of SARS-CoV-2 infection	
Asymptomatic – 1, n (%)	30 (5.0%)
Mild illness – 2, n (%)	444 (74%)
Moderate illness – 3, n (%)	109 (18%)
Severe illness – 4, n (%)	7 (1.2%)
Critical illness – 5, n (%)	6 (1.0%)
Moderate or worse COVID-19, n (%)	122 (20.5%)
Presumed virus variant	
Wild-type, n (%)	124 (21%)
Alpha, n (%)	87 (15%)
Delta, n (%)	76 (13%)
Omicron, n (%)	309 (52%)
Last eGFR before COVID-19 [mL/min/1.73m^2^], median (IQR)	48.6 (35.4, 61.8)
Body mass index before COVID-19, median (IQR)	28.0 (24.8, 30.9)
Retransplantation, n (%)	71 (12%)
Time between last Tx and COVID-19, median months (IQR)	58 (24, 110)
More than one COVID-19 infection, n (%)	83 (13.9%)
Vaccinated with at least 2 doses before COVID-19, n (%)	388 (65%)
Vaccinated with at least 3 doses before COVID-19, n (%)	305 (51%)
Charlson Comorbidity Score, median (IQR)	4.00 (2.00, 6.00)
Diabetes, n (%)	139 (23.7%)
Maintenance immunosuppression, n (%)	
Standard triple therapy (TAC + MMF/MPA + CS), n (%)	409 (69%)
Tacrolimus, n (%)	525 (88%)
Mycophenolate mofetil or Mycophenolic acid, n (%)	486 (82%)
Corticosteroids, n (%)	555 (93%)
Ciclosporin A, n (%)	42 (7%)
mTOR inhibitor, n (%)	28 (4.7%)
History of smoking, n (%)	116 (19%)

eGFR = estimated glomerular filtration rate; NIH = National Institutes of Health; MMF = mycophenolate mofetil; MPA = mycophenolic acid; IQR = interquartile range; TAC = tacrolimus; Tx = transplantation.

**Table 2.** Determinants of long COVID development in kidney transplant recipients.

	Univariable regression			Multivariable regression		
Characteristic	OR	95% CI	p-value	OR	95% CI	p-value
Male sex	0.73	0.52, 1.04	0.077	0.69	0.48, 1.0	0.047
Moderate or worse COVID-19	2.55	1.70, 3.83	< 0.001	2.49	1.58, 3.93	< 0.001
Age in years	1.02	1.01, 1.03	0.005	1.01	1.00, 1.03	0.072
Vaccinated with at least 2 doses before COVID-19	1.06	0.75, 1.52	0.7	1.33	0.68, 2.65	0.4
Body mass index before COVID-19	1.04	1.01, 1.08	0.025	1.04	1.00, 1.08	0.031
Diabetes mellitus	1.21	0.81, 1.79	0.3	0.92	0.58, 1.43	0.7
Months between COVID-19 and response	1.00	0.98, 1.03	0.7	1.00	0.96, 1.05	> 0.9
Reinfection	0.76	0.45, 1.25	0.3	0.88	0.49, 1.55	0.7
Corticosteroids	2.23	1.06, 5.29	0.047	2.83	1.24, 7.16	0.019
MMF/MPA	0.66	0.44, 1.02	0.058	0.82	0.52, 1.31	0.4
Last eGFR before COVID-19 [mL/min/1.73m^2^]	0.57	0.32, 0.99	0.049	0.86	0.47, 1.55	0.6
Months between transplantation and COVID-19	1.00	1.00, 1.00	0.4	1.00	1.00, 1.00	0.5
Targeted COVID-19 treatment (antivirals or monoclonal antibodies)	1.06	0.75, 1.52	0.7	1.11	0.69, 1,79	0.7

Table shows a multivariable logistic binary regression model and univariable association of selected variables. For all univariable associations see Supplemental Table 2. CI = confidence interval; eGFR = estimated glomerular filtration rate; MMF = mycophenolate mofetil; MPA = mycophenolic acid; OR = odds ratio.

## Supplemental material

Supplemental material Supplemental Text, Tables, and Figures.
