# Supplementary material for: Prediction and phenotyping of long COVID in kidney transplant recipients: A cross-sectional study
Source: Clin Nephrol. 2025 Mar 25;103(6):366–76. doi: 10.5414/CN111584 (PMC12097141; doi:10.5414/CN111584)
Supplement: Supplemental material [file clinnephrol-103-366_S01.pdf]

Supplementary Material

Supplementary Text 1A. Long COVID questionnaire – English translation 3
Supplementary Text 1B. Long COVID questionnaire – Czech original 5
Supplementary Table 1. Demographics of the validation cohort 7
Supplementary Table 2. Demographics of kidney transplant recipients who previously survived COVID –19 based on the reason for not participating in the survey. 8
Supplementary Table 3. Phenotype proportions based on virus variant 9
Supplementary Table 4. Phenotype proportions based on reinfections 10
Supplementary Table 5. Phenotype proportions based on vaccination status (2-dose vaccine scheme) 11
Supplementary Table 6. Univariable associations between selected variables and long COVID development. 12
Supplementary Table 7. Demographics of KTRs who developed and did not develop long COVID 13
Supplementary Table 8. Demographics of KTRs who developed cognitive phenotype of long COVID and did not develop any long COVID 15
Supplementary Table 9. Demographics of KTRs who developed cardiovascular phenotype of long COVID and did not develop any long COVID. 17
Supplementary Table 10. Demographics of KTRs who developed ophthalmic phenotype of long COVID and did not develop any long COVID. 19
Supplementary Table 11. Demographics of KTRs who developed fatigue phenotype of long COVID and did not develop any long COVID 21
Supplementary Table 12. Demographics of KTRs who developed fibromyalgia-like phenotype of long COVID and did not develop any long COVID 23
Supplementary Table 13. Demographics of KTRs who developed integument phenotype of long COVID and did not develop any long COVID. 25
Supplementary Table 14. Demographics of KTRs who developed malnutrition phenotype of long COVID and did not develop any long COVID. 27
Supplementary Table 15. Demographics of KTRs who developed psychiatric phenotype of long COVID and did not develop any long COVID. 29
Supplementary Table 16. Testing of changes in frequencies of positive answers to the individual survey questions with time from symptom onset to participating in the survey. 31
Supplementary Figure 1. Illustration of the survey on a smart phone 32
Supplementary Figure 2. Number of phenotypes optimalization. 33
Supplementary Figure 3. Heatmap of the factor loadings of responses to each question.......... 34
Supplementary Figure 4. ROC of the main model for long COVID prediction. 35
Supplementary Figure 5. Study flowchart. 36

Supplementary Figure 6. Rates of individual long COVID phenotypes in kidney transplant recipients and their overlap. 37
Supplementary Figure 7. Results of univariable logistic regression for cognitive phenotype and clinical factors. 38
Supplementary Figure 8. Results of univariable logistic regression for cardiovascular phenotype and clinical factors. 39
Supplementary Figure 9. Results of univariable logistic regression for ophthalmic phenotype and clinical factors. 40
Supplementary Figure 10. Results of univariable logistic regression for fatigue phenotype and clinical factors. 41
Supplementary Figure 11. Results of univariable logistic regression for fibromyalgia-like phenotype and clinical factors. 42
Supplementary Figure 12. Results of univariable logistic regression for integumental phenotype and clinical factors. 43
Supplementary Figure 13. Results of univariable logistic regression for malnutrition phenotype and clinical factors. 44
Supplementary Figure 14. Results of univariable logistic regression for psychiatric phenotype and clinical factors. 45
Supplementary Figure 15. Spearman correlation between factorial score in each phenotype and clinical risk factors. 46
Supplementary Figure 16. Frequency of positive replies to survey questions sorted by time from symptom onset to survey response. 47
Supplementary Figure 17. Comparison of the rates of individual long COVID phenotypes in kidney transplant recipients surveyed within the first year since COVID and more than a year since COVID-19. 48

Supplementary Text 1A. Long COVID questionnaire – English translation

1. Newly developed or worsened shortness of breath?
    a. During exertion? (none-mild-moderate-severe)
    b. At rest? (none-mild-moderate-severe)
2. Newly developed or worsened dry cough? (none-mild-moderate-severe)
3. Newly developed or worsened, or more frequent palpitations? (none-mild-moderate-severe)
4. Newly developed or worsened lower limb edema? (none-mild-moderate-severe)
5. Newly developed or worsened chest pain/feelings of pressure/tightness on the chest?
    a. During exertion? (none-mild-moderate-severe)
    b. During resting state? (none-mild-moderate-severe)
6. Newly developed or worsened joint or muscle pain? (none-mild-moderate-severe)
7. Decreased appetite? (none-mild-moderate-severe)
8. Newly developed or worsened constipation and/or diarrhea? (none-mild-moderate-severe)
9. Newly developed unexplained weight change? (none-increase-loss)
10. Newly developed or worsened of gait, coordination and/or balance? (none-mild-moderate-severe)
11. Newly developed or worsened tremor? (none-mild-moderate-severe)
12. Newly developed or worsened problems with fine motor skills (for example writing, tying shoelaces, precise manual work)? (none-mild-moderate-severe)
13. Newly developed or worsened fatigue? (none-mild-moderate-severe)
14. Newly developed or worsened feeling of faster exhaustion after previously normal activity? (none-mild-moderate-severe)
15. Newly developed or worsened problems concentrating? (none-mild-moderate-severe)
16. Newly developed or worsened problems with short-term memory? (none-mild-moderate-severe)
17. Newly developed or worsened feelings of fear and/or anxiety? (none-mild-moderate-severe)
18. Newly developed or worsened feelings of sadness and/or despair? (none-mild-moderate-severe)
19. Newly developed or worsened mood swings? (none-mild-moderate-severe)
20. Newly developed or worsened loss of interest in things around you (for example work, family, hobbies)? (none-mild-moderate-severe)
21. Newly developed or worsened sleeping problems? (none-mild-moderate-severe)
22. Newly developed or worsened quality of:
    a. Hair? (none-mild-moderate-severe)
    b. Nails? (none-mild-moderate-severe)
23. Newly occurring problems with eyesight? (none-mild-moderate-severe)
24. Newly developed or worsened feelings of dry eyes? (none-mild-moderate-severe)
25. Newly developed or worsened sensitivity to light? (none-mild-moderate-severe)
26. Newly developed or worsened problems with smell? (none-mild-moderate-severe)
27. Newly developed or worsened episodes of increased body temperature? (none-mild-moderate-severe)
28. Newly developed or worsened night sweats (to the point you have to get up and change your sheets or sleeping gown)? (yes-no)
29. Newly occurring swelling of lymph nodes in the neck, armpit or groin? (none-mild-moderate-severe)
30. Women of reproductive age only: Newly developed or worsened menstrual abnormalities? (none-mild-moderate-severe)
31. Men: Newly developed or worsened sexual dysfunctions? (none-mild-moderate-severe)
32. Newly developed muscle and/or joint morning stiffness (i.e. stiffness after waking up, limiting normal activities)? (yes/no)
33. Newly developed bilateral (at both sides) pain and/or weakness of the limb girdle (shoulders, hips)? (yes/no)
34. Would you say that your health has significantly deteriorated since having COVID-19? (yes/no)

35. Would you say that your quality of life has significantly worsened since having COVID 19? (yes/no)

Supplementary Text 1B. Long COVID questionnaire – Czech original

1. Nově vzniklé nebo zhoršující se obtíže s dušností (pocit nedostatku dechu)?
    a. při námaze? (ne-mírné-střední-těžké)
    b. v klidu? (ne-mírné-střední-těžké)
2. Nový nebo zhoršený suchý kašel? (ne-mírné-střední-těžké)
3. Nově vzniklé nebo zhoršené, případně častější bušení srdce? (ano/ne)
4. Nově vzniklé nebo zhoršení otoky dolních končetin? (ne-mírné-střední-těžké)
5. Nově vzniklé nebo zhoršující se svíravé bolesti na hrudi
    a. při námaze? (ne-mírné-střední-těžké)
    b. v klidu? (ne-mírné-střední-těžké)
6. Nově vzniklé nebo zhoršené bolesti svalů/kloubů (ne-mírné-střední-těžké)
7. Snížení chuti k jídlu? (ne-mírné-střední-těžké)
8. Nově vzniklá nebo zhoršená zácpa a/nebo průjmy (ne-mírné-střední-těžké)
9. Nová necílená změna hmotnosti? (ne-nárůst-úbytek)
10. Nově vzniklé nebo zhoršené obtíže s chůzí a koordinací pohybu a/nebo rovnováhou? (ne-mírné-střední-těžké)
11. Nově vzniklý nebo zhoršený třes? (ne-mírné-střední-těžké)
12. Nově vzniklé nebo zhoršené obtíže s jemnou motorikou (psaní, vázání tkaniček, detailní manuální činnosti) (ne-mírné-střední-těžké)
13. Nově vzniklý nebo zhoršený pocit nedostatku energie? (ne-mírné-střední-těžké)
14. Nově vzniklý nebo zhoršený pocit rychlejšího vyčerpání při dříve běžné fyzické aktivitě? (ne-mírné-střední-těžké)
15. Nově vzniklé nebo zhoršené problémy se soustředěním? (ne-mírné-střední-těžké)
16. Nově vzniklé nebo zhoršené problémy s krátkodobou pamětí? (ne-mírné-střední-těžké)
17. Nově vzniklé nebo zhoršené pocity strachu a/nebo úzkostí? (ne-mírné-střední-těžké)
18. Nově vzniklé nebo zhoršené pocity smutku a/nebo zoufalství? (ne-mírné-střední-těžké)
19. Nově vzniklé nebo zhoršené výkyvy nálad? (ne-mírné-střední-těžké)
20. Nově vzniklé nebo zhoršené pocity ztráty zájmu o věci kolem sebe (práce, rodina, koníčky)? (ne-mírné-střední-těžké)
21. Nově vzniklé nebo zhoršené problémy se spánkem? (ne-mírné-střední-těžké)
22. Nové objevení se nebo zhoršení kvality
    a. Vlasů? (ne-mírné-střední-těžké)
    b. Nehtů? (ne-mírné-střední-těžké)
23. Nově vzniklé zhoršení zraku? (ne-mírné-střední-těžké)
24. Nově vzniklá nebo zhoršená suchost očí? (ne-mírné-střední-těžké)
25. Nově vzniklá nebo zhoršená citlivost na světlo? (ne-mírné-střední-těžké)
26. Nově vzniklá nebo zhoršená porucha čichu? (ne-mírné-střední-těžké)
27. Nově vzniklé zvýšení tělesné teploty? (ne-mírné-střední-těžké)
28. Nově vzniklé nebo zhoršené noční poty (tak, že musíte měnit povlečení nebo oděv na spaní)? (ano/ne).
29. Nově vzniklé otoky lymfatických uzlin na krku, v podpaží nebo v tříslech? (ne-mírné-střední-těžké)
30. Ženy v reprodukčním věku – Nově vzniklé nebo zhoršené nepravidelnosti menstruačního cyklu? (ne-mírné-střední-těžké)
31. MUŽI – Nově vzniklé nebo zhoršené sexuální funkce? (ne-mírné-střední-těžké)
32. Nově vzniklá a pravidelná ranní ztuhlost (ztuhlost svalů a kloubů limitující běžné činnosti)? (ano/ne)
33. Nově vzniklá oboustranná bolest a/nebo slabost končetinových pletenců (ramena, kyčle)? (ano/ne)
34. Hodnotíte významné celkové zhoršení Vašeho zdravotního stavu po prodělání COVID-19? (ano/ne)

35. Hodnotíte významné celkové zhoršení Vaši kvality života po prodělání COVID-19? (ano/ne)

Supplementary Table 1. Demographics of the validation cohort

**Characteristic**	**Overall** (n = 90)
**Time between COVID-19 and response, median months (IQR)**	9 (8, 15)
**Male sex, n (%)**	55 (61%)
**Age, median (IQR)**	58 (45, 67)
**Last eGFR before COVID-19 [ml/s/1,73m2], median (IQR)**	46.8 (34.2, 60)
**Body mass index before COVID-19, median (IQR)**	27.8 (23.5, 30.1)
**Retransplantation, n (%)**	14 (16%)
**Time between last Tx and COVID-19, median months (IQR)**	49 (16, 108)
**More than one COVID-19 infection, n (%)**	15 (17%)
**Vaccinated with at least 2 doses before COVID-19, n (%)**	73 (81%)
**Vaccinated with at least 3 doses before COVID-19, n (%)**	58 (64%)
**Maintenance immunosuppression, n (%)**	
**Standard triple therapy (TAC + MMF/**MPA **+ CS), n (%)**	63 (70%)
**Tacrolimus, n (%)**	79 (88%)
**Mycophenolate mofetil or Mycophenolic acid, n (%)**	76 (84%)
**Corticosteroids, n (%)**	86 (96%)
**Ciclosporin A, n (%)**	7 (7.8%)
**mTOR inhibitor, n (%)**	2 (2.2%)

Abbreviations: eGFR, estimated glomerular filtration rate; MMF, mycophenolate mofetil; MPA, mycophenolic acid; IQR, interquartile range; TAC, tacrolimus; Tx, transplantation

Supplementary Table 2. Demographics of kidney transplant recipients who previously survived COVID –19 based on the reason for not participating in the survey.

Characteristic	Responded (n = 630)	Not filled for any reason (n = 336)	p-value	Not returned or late return (n = 248)	P-value	No consent (n=88)	P-value
**Male sex, n (%)**	392 (62%)	193 (57%)	0.15	140 (56%)	0.12	53 (60%)	0.72
**Age, median (IQR)**	56 (47, 67)	61 (50, 71)	<0.001	59 (48, 69)	0.047	66 (57,73	<0.001
**Last eGFR before COVID-19 [ml/s/1,73m2], median (IQR)**	48 (35.4, 61.2)	46.2 (33.6, 63.6)	0.33	47.4 (34.2, 64.2)	0.56	43.8 (32.4, 60.6)	0.25
**BMI before COVID-19, median (IQR)**	27.8 (24.8, 30.9)	28.1 (24.7, 31.6)	0.52	27.8 (24.6, 31,2)	0.95	29 (25.5, 31.9)	0.12
**Retransplantation, n (%)**	74 (12%)	43 (13%)	0.71	36 (15%)	0.32	7 (8%)	0.38
**Median months between last Tx and COVID-19, IQR**	58 (24, 113)	63 (26, 113)	0.29	61 (23,113)	0.63	76 (37,113)	0.13
**More than one COVID-19, n (%)**	104 (17%)	45 (13%)	0.24	32 (13%)	0.22	13 (15%)	0.8
**Vaccinated with at least 2 doses before COVID-19, n (%)**	419 (67%)	210 (63%)	0.24	156 (63%)	0.35	54 (61%)	0.4
**Vaccinated with at least 3 doses before COVID-19, n (%)**	334 (53%)	162 (48%)	0.18	119 (48%)	0.2	43 (49%)	0.54
**Maintenance immunosuppression, n (%)**							
**Standard triple therapy (TAC + MMF + CS), n (%)**	429 (69%)	226 (67%)	0.74	170 (69%)	>0.99	56 (64%)	0.42
**Tacrolimus, n (%)**	552 (88%)	284 (85%)	0.13	217 (88%)	0.87	67 (76%)	0.003
**Mycophenolate mofetil or Mycophenolic acid, n (%)**	512 (82%)	276 (82%)	0.96	200 (81%)	0.77	76 (86%)	0.37
**Corticosteroids, n (%)**	582 (93%)	315 (94%)	0.75	232 (94%)	0.88	83 (94%)	0.81
**Ciclosporin A, n (%)**	44 (7.0%)	34 (10%)	0.12	17 (6.9%)	>0.99	17 (19%)	<0.001
**mTOR inhibitor, n (%)**	28 (4.5%)	15 (4.5%)	>0.99	10 (4%)	0.92	5 (5.7%)	0.81

Abbreviations: BMI, body mass index; eGFR, estimated glomerular filtration rate; NIH, National Institutes of Health; MMF, mycophenolate mofetil; MPA, mycophenolic acid; IQR, interquartile range; TAC, tacrolimus; Tx, transplantation

Supplementary Table 3. Phenotype proportions based on virus variant

Phenotype, n (%)	Overall (n = 596)	Wild-type (n = 124)	Alpha (n = 87)	Delta (n = 76)	Omicron (n = 309)	p-value
**Any phenotype**	203 (34%)	41 (33%)	34 (39%)	24 (32%)	104 (34%)	0.74
**Cardiovascular**	36 (6.0%)	4 (3.2%)	6 (6.9%)	6 (7.9%)	20 (6.5%)	0.45
**Ophthalmic**	82 (14%)	15 (12%)	20 (23%)	9 (12%)	38 (12%)	0.085
**Cognitive**	106 (18%)	24 (19%)	19 (22%)	9 (12%)	54 (17%)	0.38
**Fibromyalgia-like**	66 (11%)	15 (12%)	12 (14%)	9 (12%)	30 (9.7%)	0.65
**Integumental**	63 (11%)	15 (12%)	11 (13%)	8 (11%)	29 (9.4%)	0.73
**Fatigue**	193 (32%)	40 (32%)	32 (37%)	23 (30%)	98 (32%)	0.80
**Psychiatric**	95 (16%)	15 (12%)	17 (20%)	12 (16%)	51 (17%)	0.51
**Malnutritional**	37 (6.2%)	4 (3.2%)	8 (9.2%)	9 (12%)	16 (5.2%)	0.050

Supplementary Table 4. Phenotype proportions based on reinfections

Phenotype, n (%)	Overall (n = 596)	COVID-19 only once (n = 531)	COVID-19 more than once (n = 83)	p-value
**Any phenotype**	203 (34%)	179 (35%)	24 (29%)	0.32
**Cardiovascular**	36 (6.0%)	33 (6.4%)	3 (3.6%)	0.46
**Ophthalmic**	82 (14%)	69 (13%)	13 (16%)	0.61
**Cognitive**	106 (18%)	96 (19%)	10 (12%)	0.16
**Fibromyalgia-like**	66 (11%)	59 (12%)	7 (8.4%)	0.57
**Integumental**	63 (11%)	57 (11%)	6 (7.2%)	0.34
**Fatigue**	193 (32%)	172 (34%)	21 (25%)	0.16
**Psychiatric**	95 (16%)	83 (16%)	12 (14%)	0.87
**Malnutritional**	37 (6.2%)	34 (6.6%)	3 (3.6%)	0.46

Supplementary Table 5. Phenotype proportions based on vaccination status (2-dose vaccine scheme)

Phenotype, n (%)	Overall (n = 596)	Full vaccination scheme not completed (n = 208)	Full vaccination scheme completed (n = 388)	p-value
**Any phenotype**	203 (34%)	69 (33%)	134 (35%)	0.79
**Cardiovascular**	36 (6.0%)	13 (6.3%)	23 (5.9%)	0.86
**Ophthalmic**	82 (14%)	31 (15%)	51 (13%)	0.62
**Cognitive**	106 (18%)	37 (18%)	69 (18%)	>0.99
**Fibromyalgia-like**	66 (11%)	26 (13%)	40 (10%)	0.41
**Integumental**	63 (11%)	26 (13%)	37 (9.5%)	0.27
**Fatigue**	193 (32%)	66 (32%)	127 (33%)	0.85
**Psychiatric**	95 (16%)	29 (14%)	66 (17%)	0.35
**Malnutritional**	37 (6.2%)	10 (4.8%)	27 (7.0%)	0.37

Supplementary Table 6. Univariable associations between selected variables and long COVID development.

Characteristic	OR	95% CI	p-value
**Months between COVID-19 and response**	1.00	0.98, 1.03	0.7
**Male sex**	0.73	0.52, 1.04	0.077
**Age in years**	1.02	1.01, 1.03	**0.005**
**COVID-19 severity**	2.05	1.52, 2.82	**<0.001**
**Moderate or worse COVID-19**	2.55	1.70, 3.83	**<0.001**
**COVID-19 period**			
**Wild type**	—	—	
**Alpha**	1.30	0.73, 2.30	0.4
**Delta**	0.93	0.50, 1.71	0.8
**Omicron**	1.03	0.66, 1.61	>0.9
**Omicron vs. others**	0.96	0.69, 1.35	0.8
**Last eGFR before COVID-19 [ml/min/1.73m^2]**	0.57	0.32, 0.99	**0.049**
**Body mass index before COVID-19**	1.04	1.01, 1.08	**0.025**
**Retransplantation**	0.92	0.53, 1.54	0.8
**Months between transplantation and COVID-19**	1.00	1.00, 1.00	0.4
**COVID-19 more than once**	0.80	0.51, 1.21	0.3
**Vaccinated with at least 2 doses before COVID-19**	0.76	0.45, 1.25	0.3
**Vaccinated with at least 3 doses before COVID-19**	1.06	0.75, 1.52	0.7
**Charlson Comorbidity Score**	0.92	0.65, 1.29	0.6
**Diabetes mellitus**	1.08	1.00, 1.16	**0.038**
**Ciclosporin A**	1.21	0.81, 1.79	0.3
**mTORi**	0.86	0.42, 1.66	0.7
**MMF/MPA**	0.91	0.39, 2.00	0.8
**Corticosteroids**	0.66	0.44, 1.02	0.058
**Tacrolimus**	2.23	1.06, 5.29	**0.047**
**Standard triple therapy (TAC + MMF + CS)**	1.01	0.61, 1.73	>0.9
**Targeted COVID-19 treatment (antivirotics or monoclonal antibodies)**	1.06	0.75, 1.52	0.7
**Last hemoglobin before COVID-19 [g/l]**	0.99	0.98, 1	0.086
**Hospitalization**	2.75	1.76, 4.32	**<0.001**

Note: Logistic binary regression was used.
Abbreviations: CI, confidence interval; eGFR, estimated glomerular filtration rate; CS, corticosteroids; MMF, mycophenolate mofetil; MPA, mycophenolic acid; TAC, tacrolimus; OR, odds ratio.

Supplementary Table 7. Demographics of KTRs who developed and did not develop long COVID

Characteristic	Overall (n = 596)	No long COVID (n = 395)	Any long COVID phenotype (n = 201)	p-value
**Median months between COVID-19 and response, IQR**	11 (9, 22)	11 (9, 22)	11 (9, 22)	0.90
**Male sex, n (%)**	364 (61%)	251 (64%)	113 (56%)	
**Median age, years (IQR)**	56 (47, 67)	55 (46, 65)	59 (49, 69)	**0.003**
**COVID-19 severity based on the NIH severity score**				**<0.001**
**Asymptomatic - 1, n (%)**	30 (5.0%)	20 (5.1%)	10 (5.0%)	
**Mild illness- 2, n (%)**	444 (74%)	314 (79%)	130 (65%)	
**Moderate illness- 3, n (%)**	109 (18%)	59 (15%)	50 (25%)	
**Severe illness - 4, n (%)**	7 (1.2%)	2 (0.5%)	5 (2.5%)	
**Critical illness- 5, n (%)**	6 (1.0%)	0 (0%)	6 (3.0%)	
**Moderate or worse COVID-19, n (%)**	122 (20%)	61 (15%)	61 (30%)	**<0.001**
**Virus variant, n (%)**				0.83
**Wild-type, n (%)**	124 (21%)	83 (21%)	41 (20%)	
**Alpha, n (%)**	87 (15%)	54 (14%)	33 (16%)	
**Delta, n (%)**	76 (13%)	52 (13%)	24 (12%)	
**Omicron, n (%)**	309 (52%)	206 (52%)	103 (51%)	
**Last eGFR before COVID-19 [ml/s/1,73m2], median (IQR)**	48.6 (35.4, 61.8)	51 (38.4, 62.4)	46.2 (31.8, 60)	**0.025**
**Body mass index before COVID-19, median (IQR)**	28.0 (24.8, 30.9)	27.6 (24.6, 30.5)	28.4 (25.3, 31.6)	**0.016**
**Retransplantation, n (%)**	71 (12%)	48 (12%)	23 (11%)	0.91
**Median months between last Tx and COVID-19, IQR**	58 (24, 110)	56 (20, 106)	65 (28, 118)	0.13
**More than one COVID-19, n (%)**	83 (14%)	59 (15%)	24 (12%)	0.38
**Vaccinated with at least 2 doses before COVID-19, n (%)**	388 (65%)	255 (65%)	133 (66%)	0.76
**Vaccinated with at least 3 doses before COVID-19, n (%)**	305 (51%)	204 (52%)	101 (50%)	0.81
**Charlson Comorbidity Score, median (IQR)**	4.00 (2.00, 6.00)	3.00 (2.00, 6.00)	4.00 (3.00, 6.00)	**0.018**
**Diabetes, n (%)**	139 (23%)	88 (22%)	51 (25%)	0.40
**Maintenance immunosuppression, n (%)**				
**Standard triple therapy (TAC + MMF + CS), n (%)**	409 (69%)	276 (70%)	133 (66%)	0.41
**Tacrolimus, n (%)**	525 (88%)	348 (88%)	177 (88%)	>0.99
**Mycophenolate mofetil or Mycophenolic acid, n (%)**	486 (82%)	331 (84%)	155 (77%)	0.061
**Corticosteroids, n (%)**	555 (93%)	362 (92%)	193 (96%)	0.068
**Ciclosporin A, n (%)**	42 (7.0%)	29 (7.3%)	13 (6.5%)	0.82

**mTOR inhibitor, n (%)**	28 (4.7%)	19 (4.8%)	9 (4.5%)	>0.99
**COVID-19 antiviral treatment (molnupiravir or remdesivir), n (%)**	283 (47%)	183 (46%)	100 (50%)	0.48
**Treatment with monoclonal antibodies, n (%)**	94 (16%)	63 (16%)	31 (15%)	0.96
**Any COVID-19 treatment (antivirotics or monocloncal antibodies), n (%)**	373 (63%)	245 (62%)	128 (64%)	0.76
**Last hemoglobin before COVID-19 [g/l]**	134 (120, 147)	135 (121, 148)	131 (118, 145)	0.1
**Hospitalization, n (%)**	95 (16%)	45 (11%)	50 (25%)	**<0.001**

Abbreviations: BMI, body mass index; CS, corticosteroids; eGFR, estimated glomerular filtration rate; KTRs, kidney transplant recipients; MMF, mycophenolate mofetil; MPA, mycophenolic acid; NIH, National Institutes of Health; IQR, interquartile range; TAC, tacrolimus; Tx, transplantation.

Supplementary Table 8. Demographics of KTRs who developed cognitive phenotype of long COVID and did not develop any long COVID

Characteristic	Cognitive phenotype (n = 106)	No long COVID (n = 395)	p-value
**Median months between COVID-19 and response, IQR**	11 (9, 22)	11 (9, 22)	0.69
**Male sex, n (%)**	62 (58%)	251 (64%)	0.34
**Median age, years (IQR)**	62 (53, 71)	55 (46, 65)	**<0.001**
**COVID-19 severity based on the NIH severity score**			**<0.001**
**Asymptomatic - 1, n (%)**	4 (3.8%)	20 (5.1%)	
**Mild illness- 2, n (%)**	67 (63%)	314 (79%)	
**Moderate illness- 3, n (%)**	27 (25%)	59 (15%)	
**Severe illness - 4, n (%)**	4 (3.8%)	2 (0.5%)	
**Critical illness- 5, n (%)**	4 (3.8%)	0 (0%)	
**Moderate or worse COVID-19, n (%)**	35 (33%)	61 (15%)	**<0.001**
**Virus variant, n (%)**			0.45
**Wild-type, n (%)**	24 (23%)	83 (21%)	
**Alpha, n (%)**	19 (18%)	54 (14%)	
**Delta, n (%)**	9 (8.5%)	52 (13%)	
**Omicron, n (%)**	54 (51%)	206 (52%)	
**Last eGFR before COVID-19 [ml/s/1,73m2], median (IQR)**	47.4 (31.8, 60)	51 (38.4, 62.4)	0.14
**Body mass index before COVID-19, median (IQR)**	29.5 (25.3, 33.3)	27.6 (24.6, 30.5)	**0.004**
**Retransplantation, n (%)**	11 (10%)	48 (12%)	0.74
**Median months between last Tx and COVID-19, IQR**	71 (33, 119)	56 (20, 106)	**0.034**
**More than one COVID-19, n (%)**	10 (9.4%)	59 (15%)	0.19
**Vaccinated with at least 2 doses before COVID-19, n (%)**	69 (65%)	255 (65%)	>0.99
**Vaccinated with at least 3 doses before COVID-19, n (%)**	55 (52%)	204 (52%)	>0.99
**Charlson Comorbidity Score, median (IQR)**	4.00 (3.00, 6.00)	3.00 (2.00, 6.00)	**<0.001**
**Diabetes, n (%)**	31 (29%)	88 (22%)	0.13
**Maintenance immunosuppression, n (%)**			
**Standard triple therapy (TAC + MMF + CS), n (%)**	70 (66%)	276 (70%)	0.52
**Tacrolimus, n (%)**	93 (88%)	348 (88%)	>0.99
**Mycophenolate mofetil or Mycophenolic acid, n (%)**	82 (77%)	331 (84%)	0.16
**Corticosteroids, n (%)**	102 (96%)	362 (92%)	0.16
**Ciclosporin A, n (%)**	7 (6.6%)	29 (7.3%)	0.96
**mTOR inhibitor, n (%)**	4 (3.8%)	19 (4.8%)	0.85

**Last hemoglobin before COVID-19 [g/l]**	137 (120, 149)	135 (121, 148)	0.86
**Hospitalization, n (%)**	28 (26%)	45 (11%)	**<0.001**
**Any COVID-19 treatment (antivirotics or monocloncal antibodies), n (%)**	63 (59%)	245 (62%)	0.71

Abbreviations: BMI, body mass index; CS, corticosteroids; eGFR, estimated glomerular filtration rate; KTRs, kidney transplant recipients; MMF, mycophenolate mofetil; MPA, mycophenolic acid; NIH, National Institutes of Health; IQR, interquartile range; TAC, tacrolimus; Tx, transplantation.

Supplementary Table 9. Demographics of KTRs who developed cardiovascular phenotype of long COVID and did not develop any long COVID

Characteristic	Cardiovascular phenotype (n = 36)	No LONG COVID (n = 395)	p-value
**Median months between COVID-19 and response, IQR**	11 (10, 21)	11 (9, 22)	0.89
**Male sex, n (%)**	19 (53%)	251 (64%)	0.20
**Median age, years (IQR)**	57 (49, 67)	55 (46, 65)	0.41
**COVID-19 severity based on the NIH severity score**			**<0.001**
**Asymptomatic - 1, n (%)**	0 (0%)	20 (5.1%)	
**Mild illness- 2, n (%)**	22 (61%)	314 (79%)	
**Moderate illness- 3, n (%)**	12 (33%)	59 (15%)	
**Severe illness - 4, n (%)**	1 (2.8%)	2 (0.5%)	
**Critical illness- 5, n (%)**	1 (2.8%)	0 (0%)	
**Moderate or worse COVID-19, n (%)**	14 (39%)	61 (15%)	**<0.001**
**Virus variant, n (%)**			0.53
**Wild-type, n (%)**	4 (11%)	83 (21%)	
**Alpha, n (%)**	6 (17%)	54 (14%)	
**Delta, n (%)**	6 (17%)	52 (13%)	
**Omicron, n (%)**	20 (56%)	206 (52%)	
**Last eGFR before COVID-19 [ml/s/1,73m2], median (IQR)**	47.4 (33.6, 65.4)	51 (38.4, 62.4)	0.53
**Body mass index before COVID-19, median (IQR)**	28.9 (24.2, 33.2)	27.6 (24.6, 30.5)	0.26
**Retransplantation, n (%)**	5 (14%)	48 (12%)	0.97
**Median months between last Tx and COVID-19, IQR**	56 (31, 101)	56 (20, 106)	0.48
**More than one COVID-19, n (%)**	3 (8.3%)	59 (15%)	0.40
**Vaccinated with at least 2 doses before COVID-19, n (%)**	23 (64%)	255 (65%)	>0.99
**Vaccinated with at least 3 doses before COVID-19, n (%)**	20 (56%)	204 (52%)	0.78
**Charlson Comorbidity Score, median (IQR)**	4.00 (2.75, 6.00)	3.00 (2.00, 6.00)	0.16
**Diabetes, n (%)**	7 (19%)	88 (22%)	0.69
**Maintenance immunosuppression, n (%)**			
**Standard triple therapy (TAC + MMF + CS), n (%)**	24 (67%)	276 (70%)	0.83
**Tacrolimus, n (%)**	30 (83%)	348 (88%)	0.57
**Mycophenolate mofetil or Mycophenolic acid, n (%)**	28 (78%)	331 (84%)	0.49
**Corticosteroids, n (%)**	35 (97%)	362 (92%)	0.39
**Ciclosporin A, n (%)**	4 (11%)	29 (7.3%)	0.63
**mTOR inhibitor, n (%)**	2 (5.6%)	19 (4.8%)	>0.99

**Last hemoglobin before COVID-19 [g/l]**	132 (118, 144)	135 (121, 148)	0.35
**Hospitalization (n%)**	14 (39%)	45 (11%)	**<0.001**
**Any COVID-19 treatment (antivirotics or monocloncal antibodies), n (%)**	24 (67%)	245 (62%)	0.71

Abbreviations: BMI, body mass index; CS, corticosteroids; eGFR, estimated glomerular filtration rate; KTRs, kidney transplant recipients; MMF, mycophenolate mofetil; MPA, mycophenolic acid; NIH, National Institutes of Health; IQR, interquartile range; TAC, tacrolimus; Tx, transplantation.

Supplementary Table 10. Demographics of KTRs who developed ophthalmic phenotype of long COVID and did not develop any long COVID

Characteristic	Ophthalmic phenotype (n = 82)	No LONG COVID (n = 395)	p-value
**Median months between COVID-19 and response, IQR**	13 (9, 22)	11 (9, 22)	0.62
**Male sex, n (%)**	42 (51%)	251 (64%)	**0.037**
**Median age, years (IQR)**	59 (49, 70)	55 (46, 65)	**0.015**
**COVID-19 severity based on the NIH severity score**			**0.004**
**Asymptomatic - 1, n (%)**	3 (3.7%)	20 (5.1%)	
**Mild illness- 2, n (%)**	55 (67%)	314 (79%)	
**Moderate illness- 3, n (%)**	20 (24%)	59 (15%)	
**Severe illness - 4, n (%)**	3 (3.7%)	2 (0.5%)	
**Critical illness- 5, n (%)**	1 (1.2%)	0 (0%)	
**Moderate or worse COVID-19, n (%)**	24 (29%)	61 (15%)	**0.003**
**Virus variant, n (%)**			0.11
**Wild-type, n (%)**	15 (18%)	83 (21%)	
**Alpha, n (%)**	20 (24%)	54 (14%)	
**Delta, n (%)**	9 (11%)	52 (13%)	
**Omicron, n (%)**	38 (46%)	206 (52%)	
**Last eGFR before COVID-19 [ml/s/1,73m2], median (IQR)**	52.2 (36.6, 66)	51 (38.4, 62.4)	0.86
**Body mass index before COVID-19, median (IQR)**	28.0 (25.0, 31.1)	27.6 (24.6, 30.5)	0.42
**Retransplantation, n (%)**	9 (11%)	48 (12%)	0.91
**Median months between last Tx and COVID-19, IQR**	63 (28, 117)	56 (20, 106)	0.37
**More than one COVID-19, n (%)**	13 (16%)	59 (15%)	0.97
**Vaccinated with at least 2 doses before COVID-19, n (%)**	51 (62%)	255 (65%)	0.78
**Vaccinated with at least 3 doses before COVID-19, n (%)**	35 (43%)	204 (52%)	0.18
**Charlson Comorbidity Score, median (IQR)**	4.00 (3.00, 6.00)	3.00 (2.00, 6.00)	**0.041**
**Diabetes, n (%)**	15 (18%)	88 (22%)	0.42
**Maintenance immunosuppression, n (%)**			
**Standard triple therapy (TAC + MMF + CS), n (%)**	54 (66%)	276 (70%)	0.56
**Tacrolimus, n (%)**	72 (88%)	348 (88%)	>0.99
**Mycophenolate mofetil or Mycophenolic acid, n (%)**	60 (73%)	331 (84%)	**0.034**
**Corticosteroids, n (%)**	79 (96%)	362 (92%)	0.22
**Ciclosporin A, n (%)**	7 (8.5%)	29 (7.3%)	0.89
**mTOR inhibitor, n (%)**	2 (2.4%)	19 (4.8%)	0.51

**Last hemoglobin before COVID-19 [g/l]**	131 (117, 144)	135 (121, 148)	0.13
**Hospitalization, n (%)**	18 (22%)	45 (11%)	0.017
**Any COVID-19 treatment (antivirotics or monocloncal antibodies), n (%)**	52 (63%)	245 (62%)	0.91

Abbreviations: BMI, body mass index; CS, corticosteroids; eGFR, estimated glomerular filtration rate; KTRs, kidney transplant recipients; MMF, mycophenolate mofetil; MPA, mycophenolic acid; NIH, National Institutes of Health; IQR, interquartile range; TAC, tacrolimus; Tx, transplantation.

Supplementary Table 11. Demographics of KTRs who developed fatigue phenotype of long COVID and did not develop any long COVID

Characteristic	Fatigue phenotype (n = 193)	No LONG COVID (n = 395)	p-value
**Median months between COVID-19 and response, IQR**	12 (9, 22)	11 (9, 22)	0.70
**Male sex, n (%)**	109 (56%)	251 (64%)	0.10
**Median age, years (IQR)**	59 (49, 69)	55 (46, 65)	**0.001**
**COVID-19 severity based on the NIH severity score**			**<0.001**
**Asymptomatic - 1, n (%)**	9 (4.7%)	20 (5.1%)	
**Mild illness- 2, n (%)**	125 (65%)	314 (79%)	
**Moderate illness- 3, n (%)**	48 (25%)	59 (15%)	
**Severe illness - 4, n (%)**	5 (2.6%)	2 (0.5%)	
**Critical illness- 5, n (%)**	6 (3.1%)	0 (0%)	
**Moderate or worse COVID-19, n (%)**	59 (31%)	61 (15%)	**<0.001**
**Virus variant, n (%)**			0.81
**Wild-type, n (%)**	40 (21%)	83 (21%)	
**Alpha, n (%)**	32 (17%)	54 (14%)	
**Delta, n (%)**	23 (12%)	52 (13%)	
**Omicron, n (%)**	98 (51%)	206 (52%)	
**Last eGFR before COVID-19 [ml/s/1,73m2], median (IQR)**	46.2 (31.8, 58.8)	51 (38.4, 62.4)	**0.023**
**Body mass index before COVID-19, median (IQR)**	28.4 (25.4, 31.6)	27.6 (24.6, 30.5)	**0.009**
**Retransplantation, n (%)**	23 (12%)	48 (12%)	>0.99
**Median months between last Tx and COVID-19, IQR**	65 (29, 116)	56 (20, 106)	0.090
**More than one COVID-19, n (%)**	21 (11%)	59 (15%)	0.22
**Vaccinated with at least 2 doses before COVID-19, n (%)**	127 (66%)	255 (65%)	0.84
**Vaccinated with at least 3 doses before COVID-19, n (%)**	96 (50%)	204 (52%)	0.73
**Charlson Comorbidity Score, median (IQR)**	4.00 (3.00, 6.00)	3.00 (2.00, 6.00)	**0.007**
**Diabetes, n (%)**	51 (26%)	88 (22%)	0.27
**Maintenance immunosuppression, n (%)**			
**Standard triple therapy (TAC + MMF + CS), n (%)**	128 (66%)	276 (70%)	0.44
**Tacrolimus, n (%)**	171 (89%)	348 (88%)	0.97
**Mycophenolate mofetil or Mycophenolic acid, n (%)**	148 (77%)	331 (84%)	**0.049**
**Corticosteroids, n (%)**	186 (96%)	362 (92%)	0.050
**Ciclosporin A, n (%)**	11 (5.7%)	29 (7.3%)	0.57
**mTOR inhibitor, n (%)**	9 (4.7%)	19 (4.8%)	>0.99

**Last hemoglobin before COVID-19 [g/l]**	132 (118,146)	135 (121, 148)	0.14
**Hospitalization, n (%)**	48 (25%)	45 (11%)	**<0.001**
**Any COVID-19 treatment (antivirotics or monocloncal antibodies), n (%)**	122 (63%)	245 (62%)	0.85

Abbreviations: BMI, body mass index; CS, corticosteroids; eGFR, estimated glomerular filtration rate; KTRs, kidney transplant recipients; MMF, mycophenolate mofetil; MPA, mycophenolic acid; NIH, National Institutes of Health; IQR, interquartile range; TAC, tacrolimus; Tx, transplantation.

Supplementary Table 12. Demographics of KTRs who developed fibromyalgia-like phenotype of long COVID and did not develop any long COVID

Characteristic	Fibromyalgia-like phenotype (n = 66)	No LONG COVID (n = 395)	p-value
**Median months between COVID-19 and response, IQR**	13 (10, 22)	11 (9, 22)	0.30
**Male sex, n (%)**	36 (55%)	251 (64%)	0.16
**Median age, years (IQR)**	57 (49, 68)	55 (46, 65)	0.085
**COVID-19 severity based on the NIH severity score**			**<0.001**
**Asymptomatic - 1, n (%)**	4 (6.1%)	20 (5.1%)	
**Mild illness- 2, n (%)**	37 (56%)	314 (79%)	
**Moderate illness- 3, n (%)**	20 (30%)	59 (15%)	
**Severe illness - 4, n (%)**	3 (4.5%)	2 (0.5%)	
**Critical illness- 5, n (%)**	2 (3.0%)	0 (0%)	
**Moderate or worse COVID-19, n (%)**	25 (38%)	61 (15%)	**<0.001**
**Virus variant, n (%)**			0.71
**Wild-type, n (%)**	15 (23%)	83 (21%)	
**Alpha, n (%)**	12 (18%)	54 (14%)	
**Delta, n (%)**	9 (14%)	52 (13%)	
**Omicron, n (%)**	30 (45%)	206 (52%)	
**Last eGFR before COVID-19 [ml/s/1,73m2], median (IQR)**	48 (31.8, 58.8)	51 (38.4, 62.4)	0.10
**Body mass index before COVID-19, median (IQR)**	29.6 (26.6, 33.3)	27.6 (24.6, 30.5)	**<0.001**
**Retransplantation, n (%)**	9 (14%)	48 (12%)	0.89
**Median months between last Tx and COVID-19, IQR**	57 (29, 107)	56 (20, 106)	0.71
**More than one COVID-19, n (%)**	7 (11%)	59 (15%)	0.46
**Vaccinated with at least 2 doses before COVID-19, n (%)**	40 (61%)	255 (65%)	0.63
**Vaccinated with at least 3 doses before COVID-19, n (%)**	29 (44%)	204 (52%)	0.30
**Charlson Comorbidity Score, median (IQR)**	4.00 (2.00, 6.00)	3.00 (2.00, 6.00)	0.18
**Diabetes, n (%)**	19 (29%)	88 (22%)	0.25
**Maintenance immunosuppression, n (%)**			
**Standard triple therapy (TAC + MMF + CS), n (%)**	50 (76%)	276 (70%)	0.41
**Tacrolimus, n (%)**	57 (86%)	348 (88%)	0.84
**Mycophenolate mofetil or Mycophenolic acid, n (%)**	58 (88%)	331 (84%)	0.51
**Corticosteroids, n (%)**	64 (97%)	362 (92%)	0.21
**Ciclosporin A, n (%)**	6 (9.1%)	29 (7.3%)	0.81
**mTOR inhibitor, n (%)**	0 (0%)	19 (4.8%)	0.14

**Last hemoglobin before COVID-19 [g/l]**	133 (119, 140)	135 (121, 148)	0.13
**Hospitalization, n (%)**	22 (33%)	45 (11%)	**<0.001**
**Any COVID-19 treatment (antivirotics or monocloncal antibodies), n (%)**	37 (56%)	245 (62%)	0.43

Abbreviations: BMI, body mass index; CS, corticosteroids; eGFR, estimated glomerular filtration rate; KTRs, kidney transplant recipients; MMF, mycophenolate mofetil; MPA, mycophenolic acid; NIH, National Institutes of Health; IQR, interquartile range; TAC, tacrolimus; Tx, transplantation.

Supplementary Table 13. Demographics of KTRs who developed integument phenotype of long COVID and did not develop any long COVID

Characteristic	Integumental phenotype (n = 63)	No LONG COVID (n = 395)	p-value
**Median months between COVID-19 and response, IQR**	12 (9, 23)	11 (9, 22)	0.54
**Male sex, n (%)**	26 (41%)	251 (64%)	**<0.001**
**Median age, years (IQR)**	59 (49, 70)	55 (46, 65)	0.076
**COVID-19 severity based on the NIH severity score**			**<0.001**
**Asymptomatic - 1, n (%)**	4 (6.3%)	20 (5.1%)	
**Mild illness- 2, n (%)**	35 (56%)	314 (79%)	
**Moderate illness- 3, n (%)**	19 (30%)	59 (15%)	
**Severe illness - 4, n (%)**	2 (3.2%)	2 (0.5%)	
**Critical illness- 5, n (%)**	3 (4.8%)	0 (0%)	
**Moderate or worse COVID-19, n (%)**	24 (38%)	61 (15%)	**<0.001**
**Virus variant, n (%)**			0.76
**Wild-type, n (%)**	15 (24%)	83 (21%)	
**Alpha, n (%)**	11 (17%)	54 (14%)	
**Delta, n (%)**	8 (13%)	52 (13%)	
**Omicron, n (%)**	29 (46%)	206 (52%)	
**Last eGFR before COVID-19 [ml/s/1,73m2], median (IQR)**	46.8 (28.8, 61.2)	51 (38.4, 62.4)	0.12
**Body mass index before COVID-19, median (IQR)**	28.0 (24.0, 32.7)	27.6 (24.6, 30.5)	0.46
**Retransplantation, n (%)**	8 (13%)	48 (12%)	>0.99
**Median months between last Tx and COVID-19, IQR**	77 (29, 106)	56 (20, 106)	0.23
**More than one COVID-19, n (%)**	6 (9.5%)	59 (15%)	0.34
**Vaccinated with at least 2 doses before COVID-19, n (%)**	37 (59%)	255 (65%)	0.45
**Vaccinated with at least 3 doses before COVID-19, n (%)**	31 (49%)	204 (52%)	0.82
**Charlson Comorbidity Score, median (IQR)**	4.00 (3.00, 7.00)	3.00 (2.00, 6.00)	**0.025**
**Diabetes, n (%)**	15 (24%)	88 (22%)	0.79
**Maintenance immunosuppression, n (%)**			
**Standard triple therapy (TAC + MMF + CS), n (%)**	42 (67%)	276 (70%)	0.71
**Tacrolimus, n (%)**	55 (87%)	348 (88%)	>0.99
**Mycophenolate mofetil or Mycophenolic acid, n (%)**	49 (78%)	331 (84%)	0.32
**Corticosteroids, n (%)**	61 (97%)	362 (92%)	0.24
**Ciclosporin A, n (%)**	2 (3.2%)	29 (7.3%)	0.34
**mTOR inhibitor, n (%)**	3 (4.8%)	19 (4.8%)	>0.99

**Last hemoglobin before COVID-19 [g/l]**	125 (113,142)	135 (121, 148)	0.003
**Hospitalization n (%)**	16 (25%)	45 (11%)	0.005
**Any COVID-19 treatment (antivirotics or monocloncal antibodies), n (%)**	40 (63%)	245 (62%)	0.93

Abbreviations: BMI, body mass index; CS, corticosteroids; eGFR, estimated glomerular filtration rate; KTRs, kidney transplant recipients; MMF, mycophenolate mofetil; MPA, mycophenolic acid; NIH, National Institutes of Health; IQR, interquartile range; TAC, tacrolimus; Tx, transplantation.

Supplementary Table 14. Demographics of KTRs who developed malnutrition phenotype of long COVID and did not develop any long COVID

Characteristic	Malnutrition phenotype (n = 37)	No LONG COVID (n = 395)	p-value
**Median months between COVID-19 and response, IQR**	13 (10, 21)	11 (9, 22)	0.45
**Male sex, n (%)**	17 (46%)	251 (64%)	**0.035**
**Median age, years (IQR)**	61 (49, 71)	55 (46, 65)	0.073
**COVID-19 severity based on the NIH severity score**			**0.024**
**Asymptomatic - 1, n (%)**	3 (8.1%)	20 (5.1%)	
**Mild illness- 2, n (%)**	21 (57%)	314 (79%)	
**Moderate illness- 3, n (%)**	12 (32%)	59 (15%)	
**Severe illness - 4, n (%)**	0 (0%)	2 (0.5%)	
**Critical illness- 5, n (%)**	1 (2.7%)	0 (0%)	
**Moderate or worse COVID-19, n (%)**	13 (35%)	61 (15%)	**0.002**
**Virus variant, n (%)**			0.080
**Wild-type, n (%)**	4 (11%)	83 (21%)	
**Alpha, n (%)**	8 (22%)	54 (14%)	
**Delta, n (%)**	9 (24%)	52 (13%)	
**Omicron, n (%)**	16 (43%)	206 (52%)	
**Last eGFR before COVID-19 [ml/s/1,73m2], median (IQR)**	45 (33, 58.8)	51 (38.4, 62.4)	0.21
**Body mass index before COVID-19, median (IQR)**	28.0 (23.7, 31.6)	27.6 (24.6, 30.5)	0.75
**Retransplantation, n (%)**	3 (8.1%)	48 (12%)	0.64
**Median months between last Tx and COVID-19, IQR**	71 (33, 130)	56 (20, 106)	0.12
**More than one COVID-19, n (%)**	3 (8.1%)	59 (15%)	0.37
**Vaccinated with at least 2 doses before COVID-19, n (%)**	27 (73%)	255 (65%)	0.40
**Vaccinated with at least 3 doses before COVID-19, n (%)**	18 (49%)	204 (52%)	0.86
**Charlson Comorbidity Score, median (IQR)**	4.00 (3.00, 6.00)	3.00 (2.00, 6.00)	0.12
**Diabetes, n (%)**	7 (19%)	88 (22%)	0.64
**Maintenance immunosuppression, n (%)**			
**Standard triple therapy (TAC + MMF + CS), n (%)**	23 (62%)	276 (70%)	0.43
**Tacrolimus, n (%)**	34 (92%)	348 (88%)	0.67
**Mycophenolate mofetil or Mycophenolic acid, n (%)**	26 (70%)	331 (84%)	0.064
**Corticosteroids, n (%)**	35 (95%)	362 (92%)	0.75
**Ciclosporin A, n (%)**	2 (5.4%)	29 (7.3%)	0.92
**mTOR inhibitor, n (%)**	2 (5.4%)	19 (4.8%)	>0.99

**Last hemoglobin before COVID-19 [g/l]**	131 (121, 142)	135 (121, 148)	0.32
**Hospitalization, n (%)**	7 (19%)	45 (11%)	0.28
**Any COVID-19 treatment (antivirotics or monocloncal antibodies), n (%)**	19 (51%)	245 (62%)	0.27

Abbreviations: BMI, body mass index; CS, corticosteroids; eGFR, estimated glomerular filtration rate; KTRs, kidney transplant recipients; MMF, mycophenolate mofetil; MPA, mycophenolic acid; NIH, National Institutes of Health; IQR, interquartile range; TAC, tacrolimus; Tx, transplantation.

Supplementary Table 15. Demographics of KTRs who developed psychiatric phenotype of long COVID and did not develop any long COVID

Characteristic	Psychiatric phenotype (n = 95)	No LONG COVID (n = 325)	p-value
**Median months between COVID-19 and response, IQR**	11 (9, 21)	11 (9, 22)	0.51
**Male sex, n (%)**	44 (46%)	251 (64%)	**0.002**
**Median age, years (IQR)**	57 (49, 67)	55 (46, 65)	0.20
**COVID-19 severity based on the NIH severity score**			**<0.001**
**Asymptomatic - 1, n (%)**	3 (3.2%)	20 (5.1%)	
**Mild illness- 2, n (%)**	63 (66%)	314 (79%)	
**Moderate illness- 3, n (%)**	26 (27%)	59 (15%)	
**Severe illness - 4, n (%)**	2 (2.1%)	2 (0.5%)	
**Critical illness- 5, n (%)**	1 (1.1%)	0 (0%)	
**Moderate or worse COVID-19, n (%)**	29 (31%)	61 (15%)	**<0.001**
**Virus variant, n (%)**			0.56
**Wild-type, n (%)**	15 (16%)	83 (21%)	
**Alpha, n (%)**	17 (18%)	54 (14%)	
**Delta, n (%)**	12 (13%)	52 (13%)	
**Omicron, n (%)**	51 (54%)	206 (52%)	
**Last eGFR before COVID-19 [ml/s/1,73m2], median (IQR)**	48 (32.4, 61.2)	51 (38.4, 62.4)	0.17
**Body mass index before COVID-19, median (IQR)**	28.6 (25.1, 32.0)	27.6 (24.6, 30.5)	**0.035**
**Retransplantation, n (%)**	11 (12%)	48 (12%)	>0.99
**Median months between last Tx and COVID-19, IQR**	83 (32, 118)	56 (20, 106)	**0.042**
**More than one COVID-19, n (%)**	12 (13%)	59 (15%)	0.68
**Vaccinated with at least 2 doses before COVID-19, n (%)**	66 (69%)	255 (65%)	0.43
**Vaccinated with at least 3 doses before COVID-19, n (%)**	49 (52%)	204 (52%)	>0.99
**Charlson Comorbidity Score, median (IQR)**	4.00 (2.00, 5.00)	3.00 (2.00, 6.00)	0.46
**Diabetes, n (%)**	21 (22%)	88 (22%)	0.97
**Maintenance immunosuppression, n (%)**			
**Standard triple therapy (TAC + MMF + CS), n (%)**	67 (71%)	276 (70%)	>0.99
**Tacrolimus, n (%)**	88 (93%)	348 (88%)	0.28
**Mycophenolate mofetil or Mycophenolic acid, n (%)**	73 (77%)	331 (84%)	0.15
**Corticosteroids, n (%)**	92 (97%)	362 (92%)	0.13
**Ciclosporin A, n (%)**	5 (5.3%)	29 (7.3%)	0.62
**mTOR inhibitor, n (%)**	5 (5.3%)	19 (4.8%)	>0.99

**Last hemoglobin before COVID-19 [g/l]**	130 (115, 143)	135 (121, 148)	0.024
**Hospitalization, n (%)**	26 (27%)	45 (11%)	**<0.001**
**Any COVID-19 treatment (antivirotics or monocloncal antibodies), n (%)**	67 (71%)	245 (62%)	0.15

Abbreviations: BMI, body mass index; CS, corticosteroids; eGFR, estimated glomerular filtration rate; KTRs, kidney transplant recipients; MMF, mycophenolate mofetil; MPA, mycophenolic acid; NIH, National Institutes of Health; IQR, interquartile range; TAC, tacrolimus; Tx, transplantation.

Supplementary Table 16. Testing of changes in frequencies of positive answers to the individual survey questions with time from symptom onset to participating in the survey.

Symptom	p-value	p-value adjusted for multiple testing*
Dyspnea during exertion	0.63	1
Resting dyspnea	0.26	1
Cough	0.56	1
Palpitations	0.35	1
Edema	0.33	1
Chest pain at rest	0.77	1
Chest pain during exertion	0.071	1
Joint/muscle pain	0.004	0.13
Decreased appetite	0.011	0.39
Changes in bowel movements	0.16	1
Weight change	0.42	1
Gait, balance, coordination	0.68	1
Tremor	0.76	1
Fine motor skills	0.06	1
Fatigue	0.75	1
Exhaustion after exertion	0.64	1
Concentration	0.25	1
Memory	0.47	1
Fear/anxiety	0.26	1
Sadness/despair	0.77	1
Mood swings	0.2	1
Anhedonia	0.28	1
Sadness/despair	0.77	1
Sleep	0.92	1
Hair quality	0.71	1
Nail quality	0.65	1
Eyesight	0.12	1
Dry eyes	0.065	1
Photophobia	0.64	1
Parosmia	0.35	1
Increased temperature	0.46	1
Night sweats	0.55	1
Lymphadenopathy	0.76	1
Menstrual abnormalities	0.37	1
Sexual dysfunction in men	0.37	1
Muscle/join morning stiffness	0.38	1
Girdle pain/weakness	0.35	1

The p-value was calculated by the Fischers exact test.
*The correction for multiple testing was performed by the Bonferroni correction.

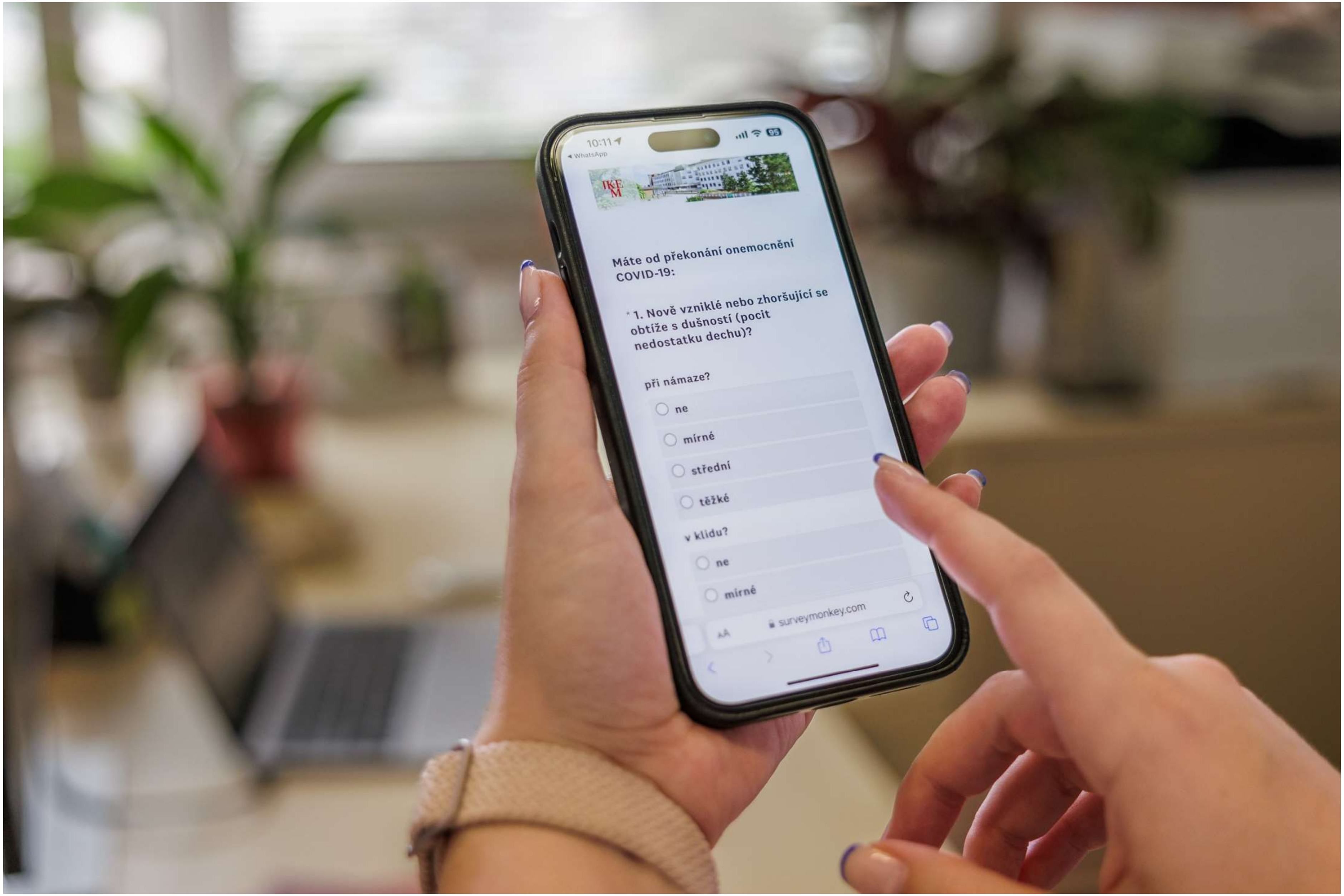

Supplementary Figure 1. Illustration of the survey on a smart phone

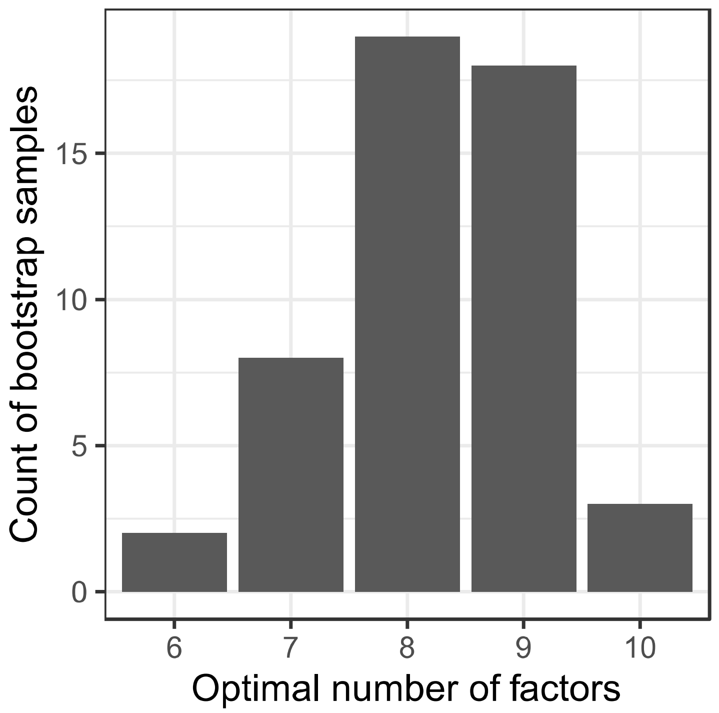

Supplementary Figure 2. Number of phenotypes optimalization.
The optimal number of selected factors was determined by bootstrapping (50 iterations); the number of factors that occurred most frequently among bootstrapped datasets was chosen as the optimal number.

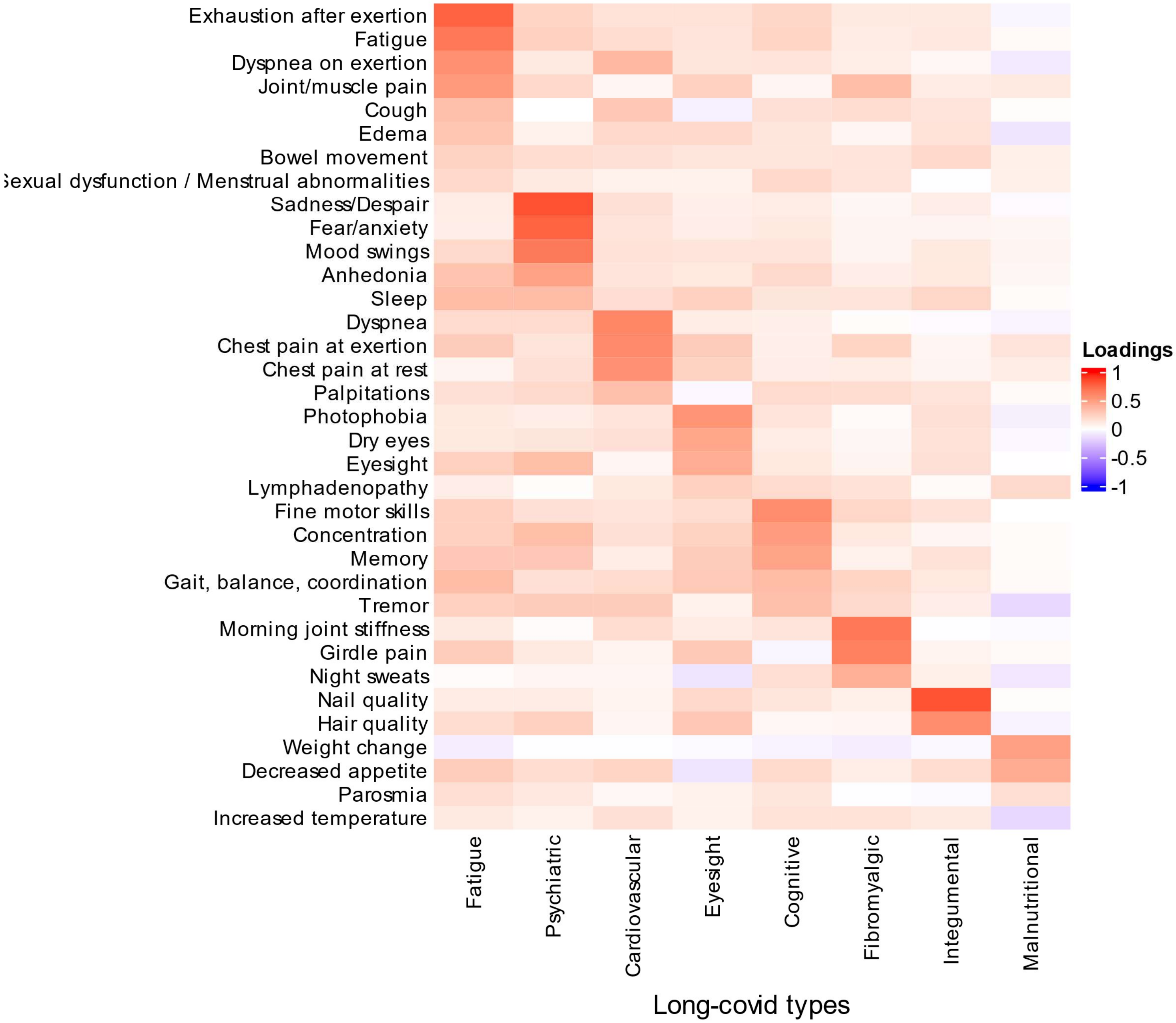

Supplementary Figure 3. Heatmap of the factor loadings of responses to each question.
Questions with factor loadings above 0.3 were clustered into factors based on the highest factor loadings.

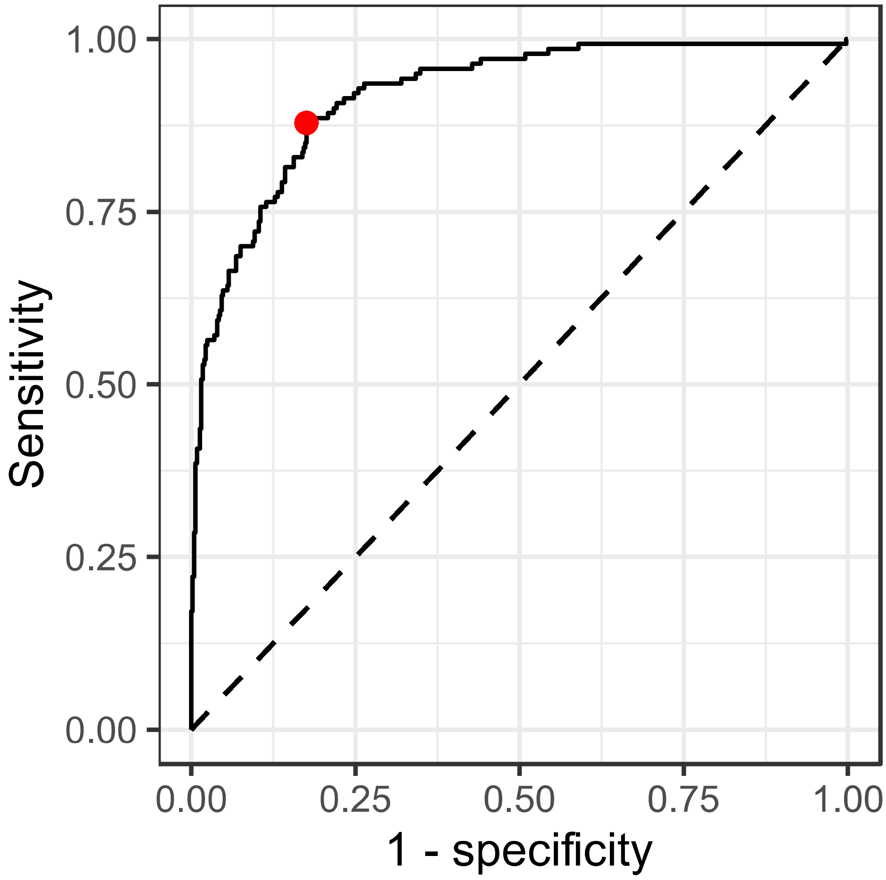

Supplementary Figure 4. ROC of the main model for long COVID prediction. AUC = 91.95%.

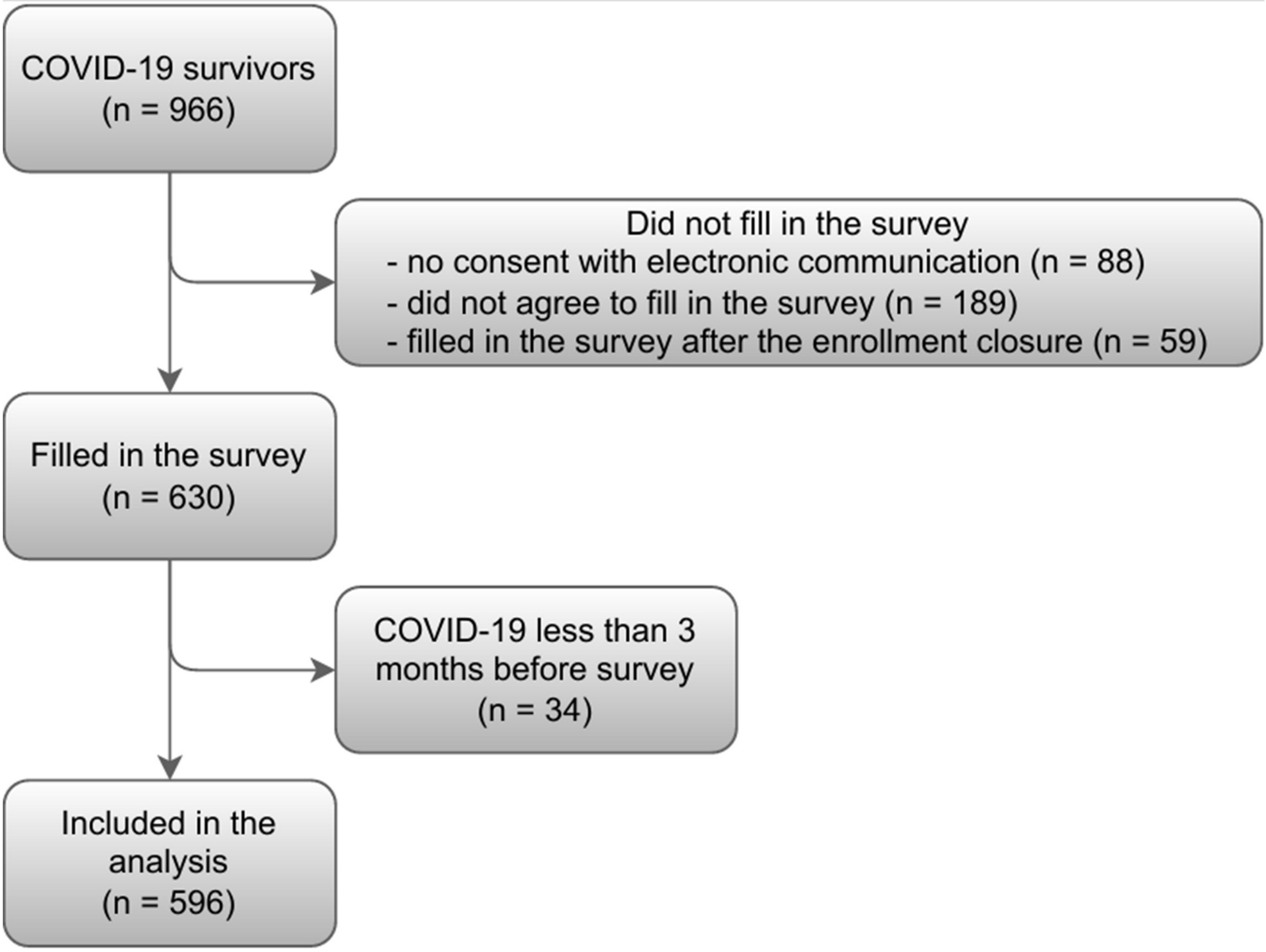

Supplementary Figure 5. Study flowchart.

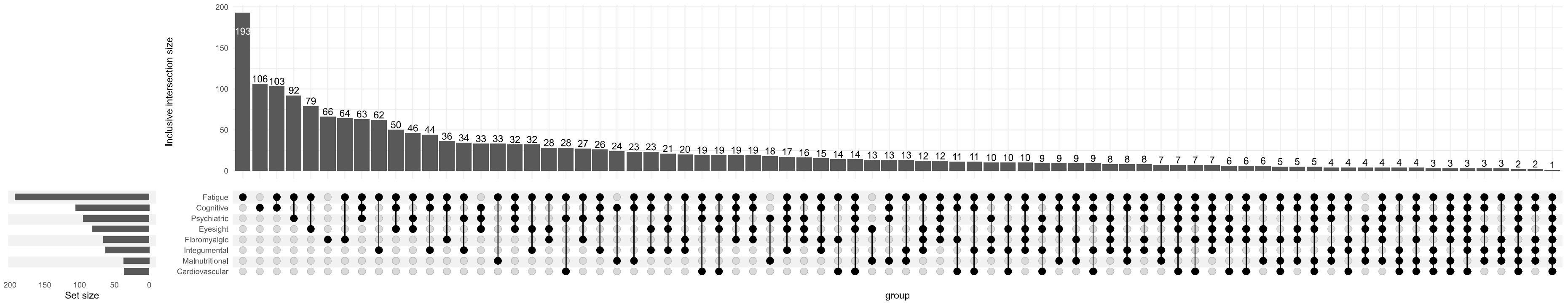

Supplementary Figure 6. Rates of individual long COVID phenotypes in kidney transplant recipients and their overlap.
Eight long COVID phenotypes were identified. Each patient can be classified by one or more long COVID phenotypes; the overlap is shown in the upset plot. The rates in the upset plot were computed as inclusive intersections, meaning that a specific combination of phenotypes (one bar in the upset plot) might be a subset of larger set of phenotypes. For example, 103 patients have phenotype Fatigue and Cognitive, but inclusive intersection means that these patients might have additional phenotypes.

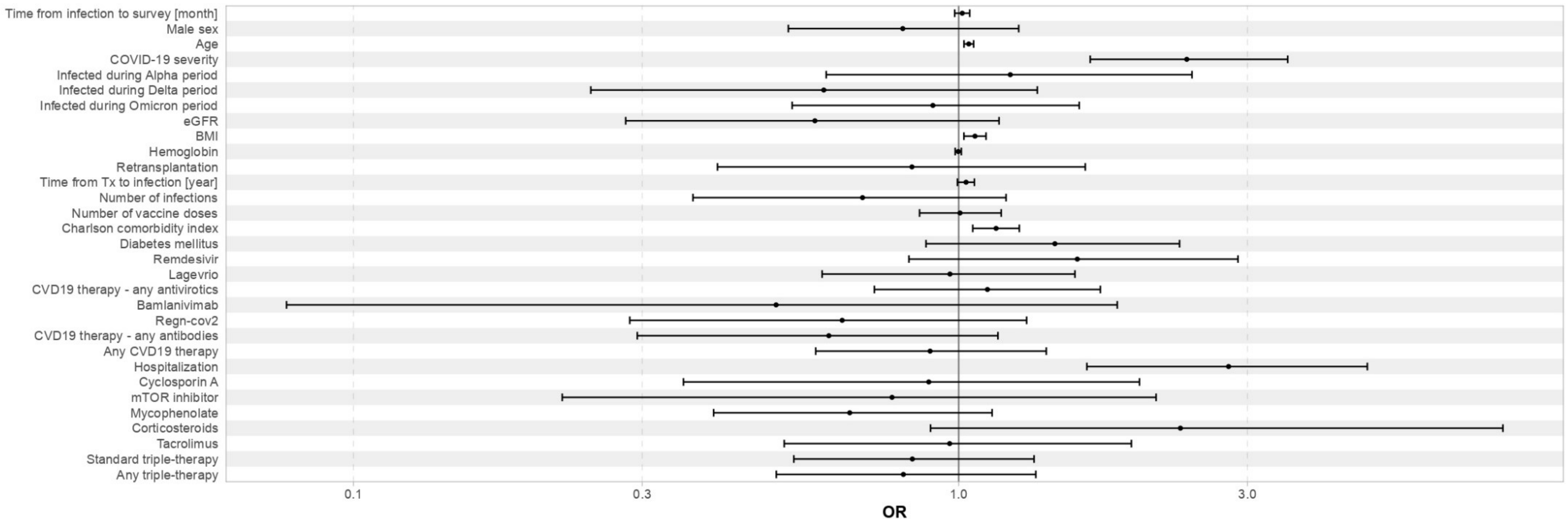

Supplementary Figure 7. Results of univariable logistic regression for cognitive phenotype and clinical factors.
Note: Vertical line indicates OR (odds ratio) = 1, dot is the calculated OR, whisks indicate 95% confidence intervals. The x-axis is log-transformed
Abbreviations: eGFR, estimated glomerular filtration rate; BMI, body mass index; Tx, transplantation; mTORi, inhibitor of mechanistic target of rapamycin.

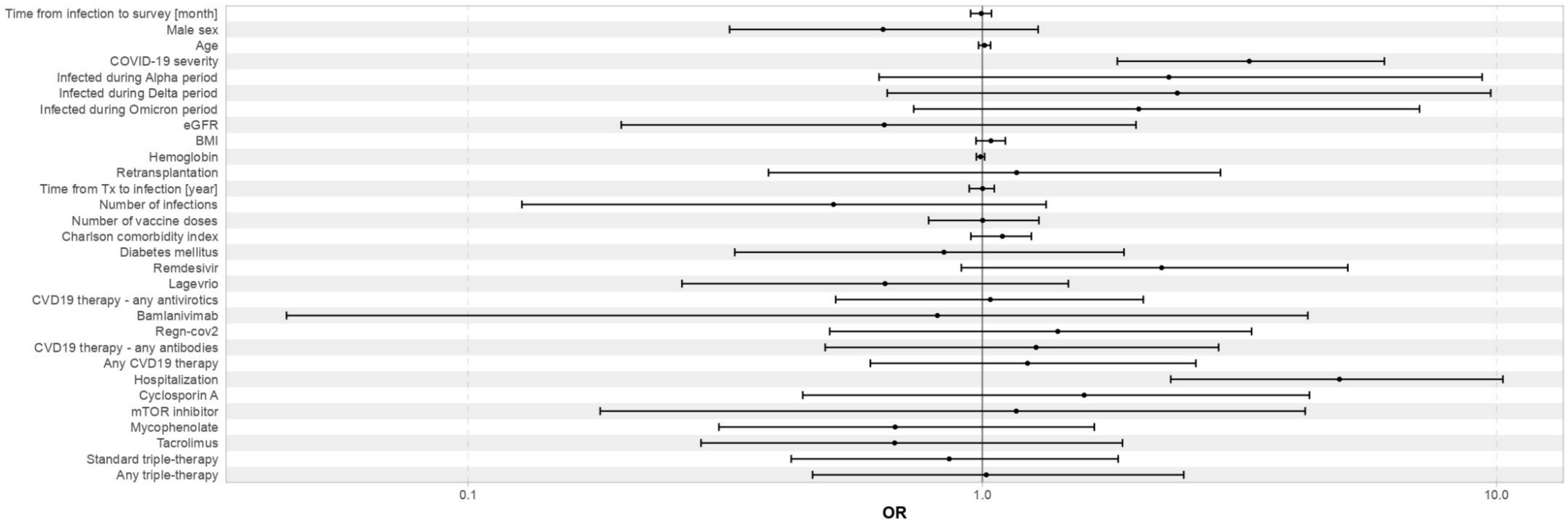

Supplementary Figure 8. Results of univariable logistic regression for cardiovascular phenotype and clinical factors.
Note: Vertical line indicates OR (odds ratio) = 1, dot is the calculated OR, whisks indicate 95% confidence intervals. The x-axis is log-transformed
Abbreviations: eGFR, estimated glomerular filtration rate; BMI, body mass index; Tx, transplantation; mTORi, inhibitor of mechanistic target of rapamycin.

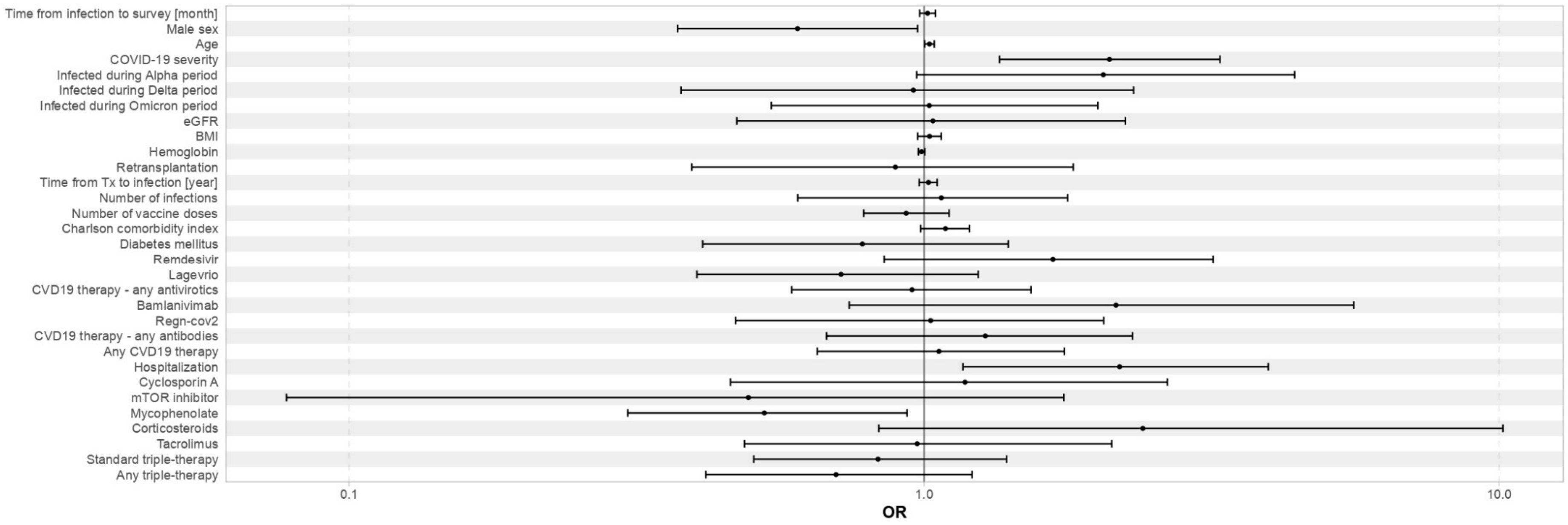

Supplementary Figure 9. Results of univariable logistic regression for ophthalmic phenotype and clinical factors.
Note: Vertical line indicates OR (odds ratio) = 1, dot is the calculated OR, whisks indicate 95% confidence intervals. The x-axis is log-transformed
Abbreviations: eGFR, estimated glomerular filtration rate; BMI, body mass index; Tx, transplantation; mTORi, inhibitor of mechanistic target of rapamycin.

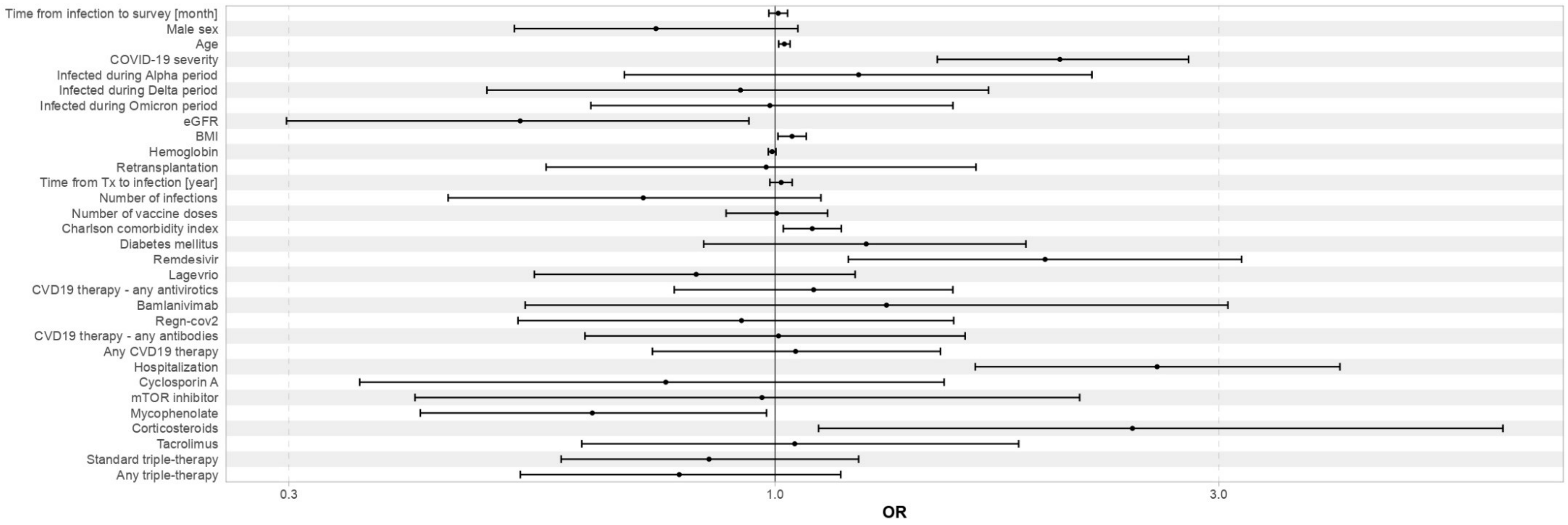

Supplementary Figure 10. Results of univariable logistic regression for fatigue phenotype and clinical factors.
Note: Vertical line indicates OR (odds ratio) = 1, dot is the calculated OR, whisks indicate 95% confidence intervals. The x-axis is log-transformed
Abbreviations: eGFR, estimated glomerular filtration rate; BMI, body mass index; Tx, transplantation; mTORi, inhibitor of mechanistic target of rapamycin.

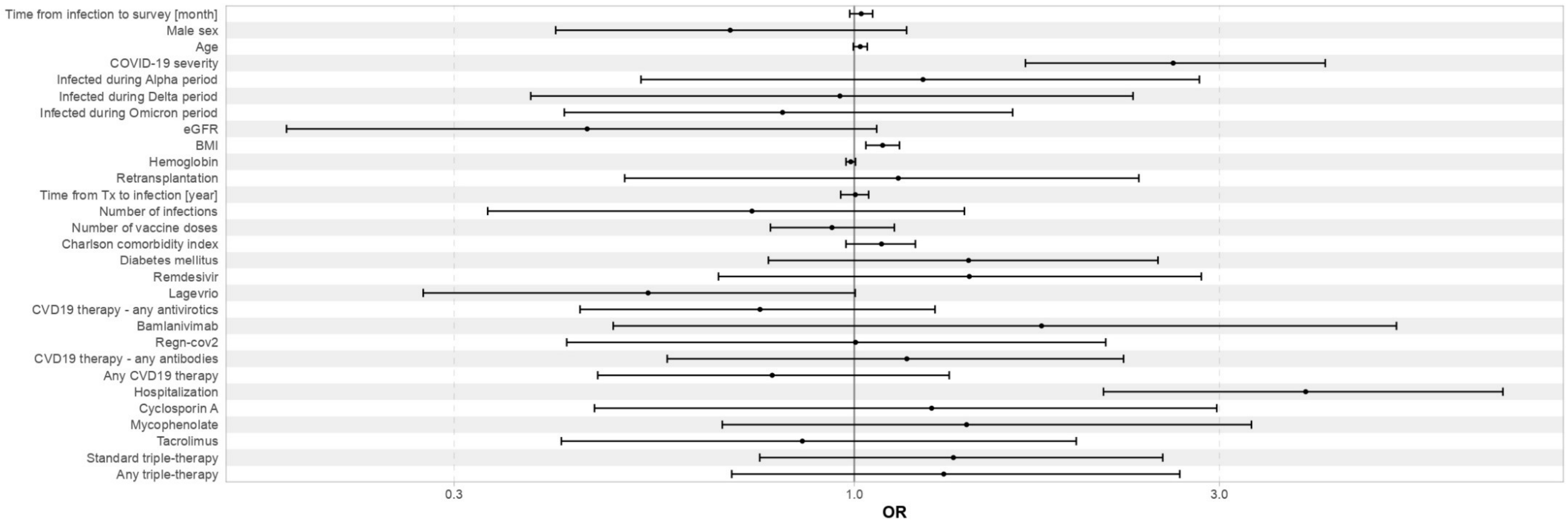

Supplementary Figure 11. Results of univariable logistic regression for fibromyalgia-like phenotype and clinical factors.
Note: Vertical line indicates OR (odds ratio) = 1, dot is the calculated OR, whisks indicate 95% confidence intervals. The x-axis is log-transformed
Abbreviations: eGFR, estimated glomerular filtration rate; BMI, body mass index; Tx, transplantation; mTORi, inhibitor of mechanistic target of rapamycin.

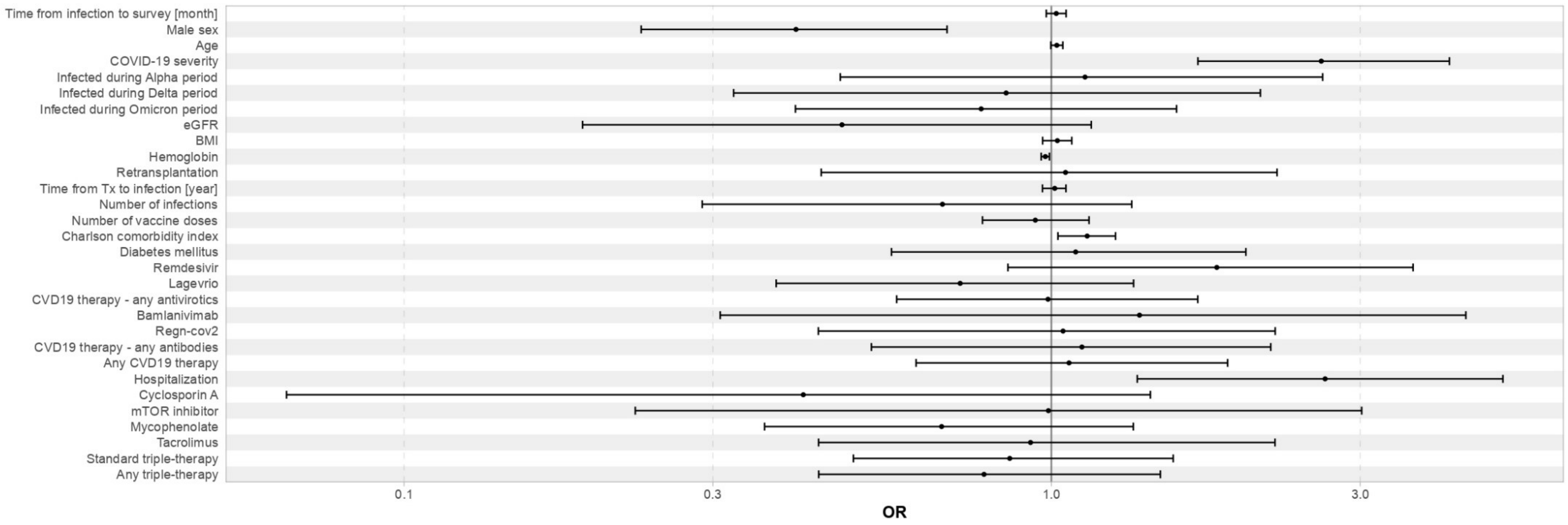

Supplementary Figure 12. Results of univariable logistic regression for integumental phenotype and clinical factors.
Note: Vertical line indicates OR (odds ratio) = 1, dot is the calculated OR, whisks indicate 95% confidence intervals. The x-axis is log-transformed
Abbreviations: eGFR, estimated glomerular filtration rate; BMI, body mass index; Tx, transplantation; mTORi, inhibitor of mechanistic target of rapamycin.

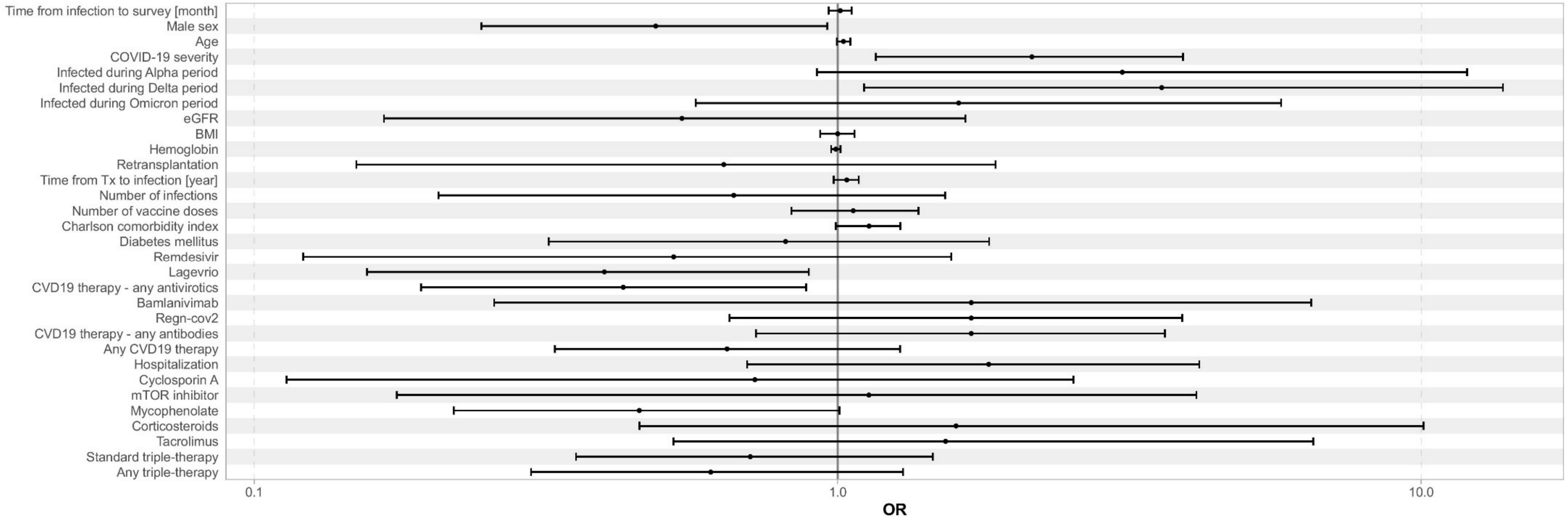

Supplementary Figure 13. Results of univariable logistic regression for malnutrition phenotype and clinical factors.
Note: Vertical line indicates OR (odds ratio) = 1, dot is the calculated OR, whisks indicate 95% confidence intervals. The x-axis is log-transformed
Abbreviations: eGFR, estimated glomerular filtration rate; BMI, body mass index; Tx, transplantation; mTORi, inhibitor of mechanistic target of rapamycin.

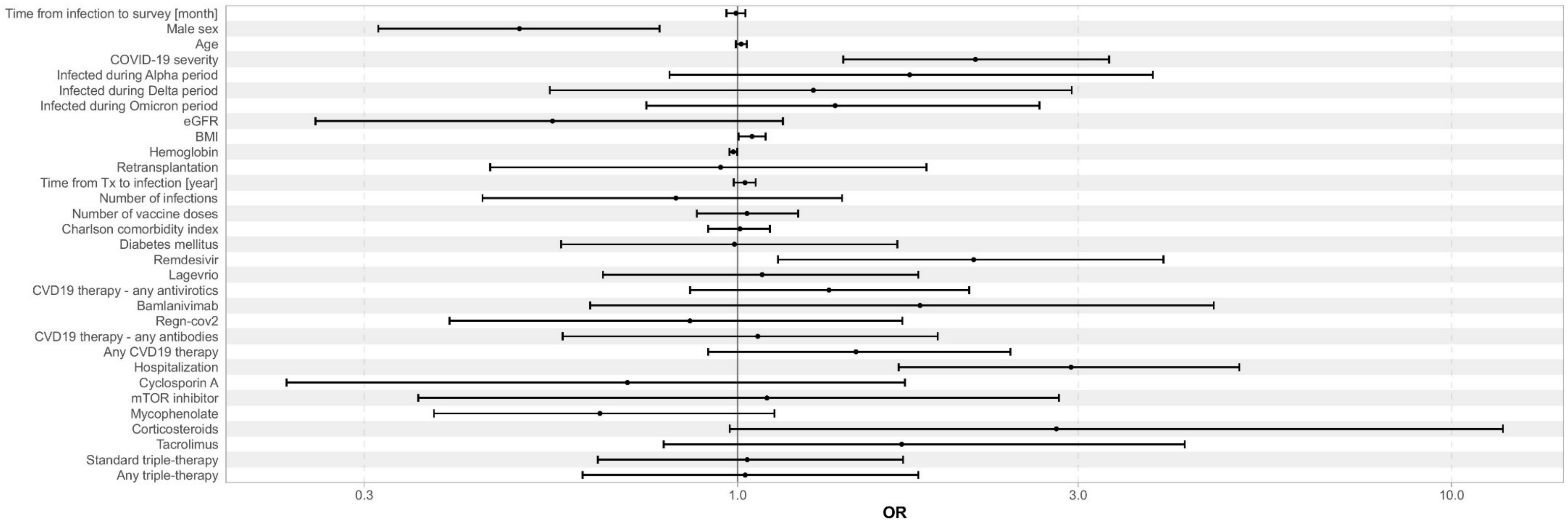

Supplementary Figure 14. Results of univariable logistic regression for psychiatric phenotype and clinical factors.
Note: Vertical line indicates OR (odds ratio) = 1, dot is the calculated OR, whisks indicate 95% confidence intervals. The x-axis is log-transformed
Abbreviations: eGFR, estimated glomerular filtration rate; BMI, body mass index; Tx, transplantation; mTORi, inhibitor of mechanistic target of rapamycin.

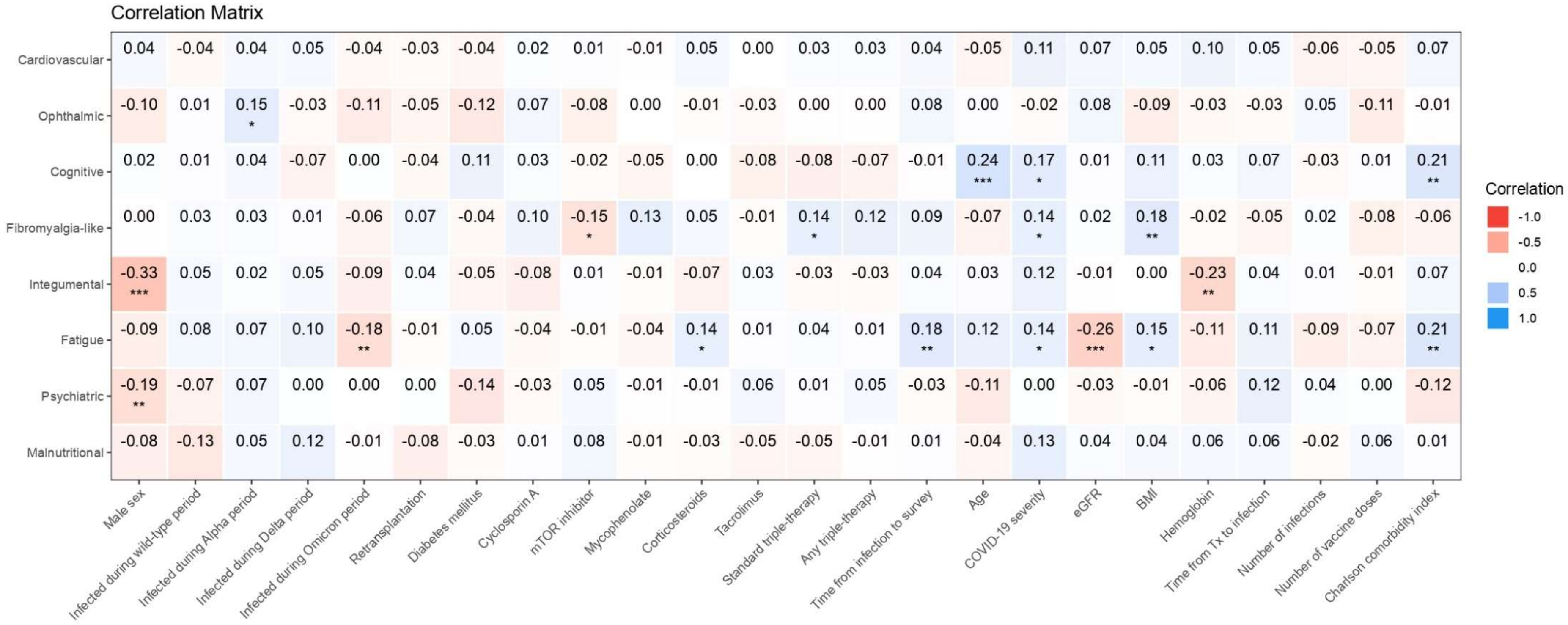

Supplementary Figure 15. Spearman correlation between factorial score in each phenotype and clinical risk factors.
The color indicates the direction of correlation (red is positive correlation, blue is negative correlation), the intensity of the color indicates correlation coefficient that is also calculated in all cells. Asterisks (* - ***) indicate statistical significance.

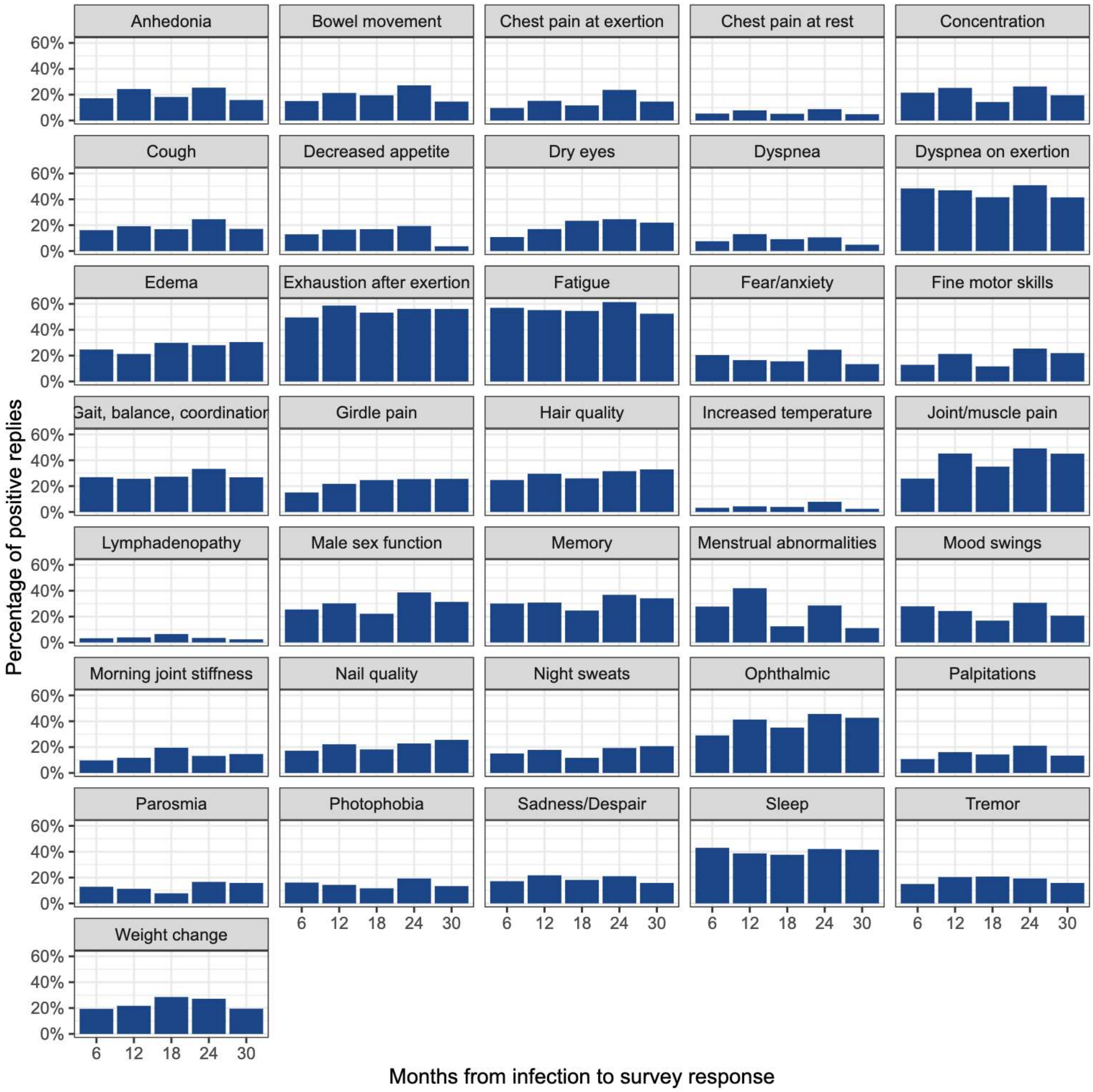

Supplementary Figure 16. Frequency of positive replies to survey questions sorted by time from symptom onset to survey response.

The X axis denotes the category, which is based on the number of months since symptom onset to survey response (i.e., 3-6 months, 7-12 months, 13-18 months, 19-24 months, 25-30 months). The Y axis denotes the percentage of kidney transplant recipients who answered positively to the question.

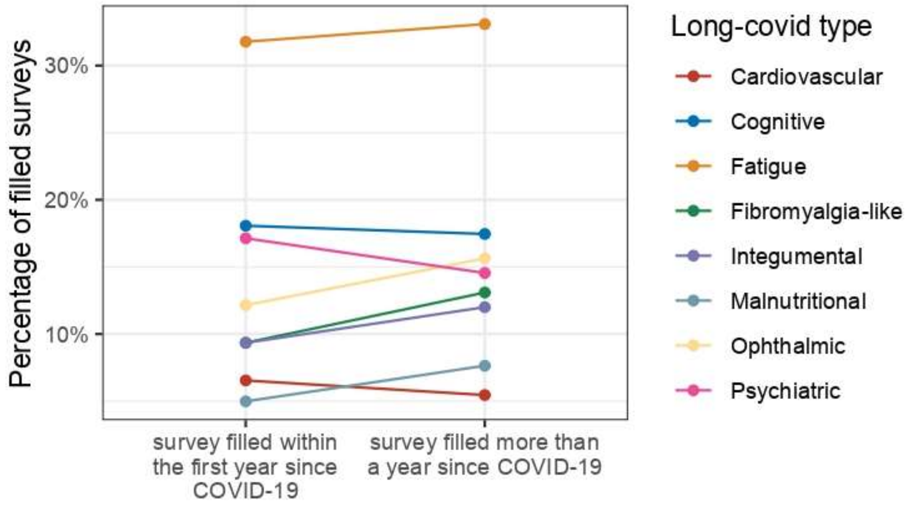

Supplementary Figure 17. Comparison of the rates of individual long COVID phenotypes in kidney transplant recipients surveyed within the first year since COVID and more than a year since COVID-19.
The percentages of all identified long COVID phenotypes were similar within the first year and later – (p = 0.61 for the cardiovascular phenotype, p = 0.91 for the cognitive phenotype, p = 0.23 for ophthalmic phenotype, p = 0.79 for the fatigue phenotype, p = 0.15 for the fibromyalgia-like phenotype, p = 0.34 for the integumental phenotype, p = 0.23 for the malnutritional phenotype, and p = 0.43 for the psychiatric phenotype).
